# Strategies for Conditional Regulation of Proteins

**DOI:** 10.1021/jacsau.2c00654

**Published:** 2023-01-26

**Authors:** Karthik Nadendla, Grant G. Simpson, Julie Becher, Toby Journeaux, Mar Cabeza-Cabrerizo, Gonçalo J. L. Bernardes

**Affiliations:** † Yusuf Hamied Department of Chemistry, University of Cambridge, CB2 1EW, Cambridge, U.K.; ‡ Instituto de Medicina Molecular João Lobo Antunes, Faculdade de Medicina, Universidade de Lisboa, 1649-028 Lisboa, Portugal

**Keywords:** Proteins and peptides, conditional regulation, covalent protein regulation, noncovalent protein regulation, conformational protein regulation, protein compartmentalization

## Abstract

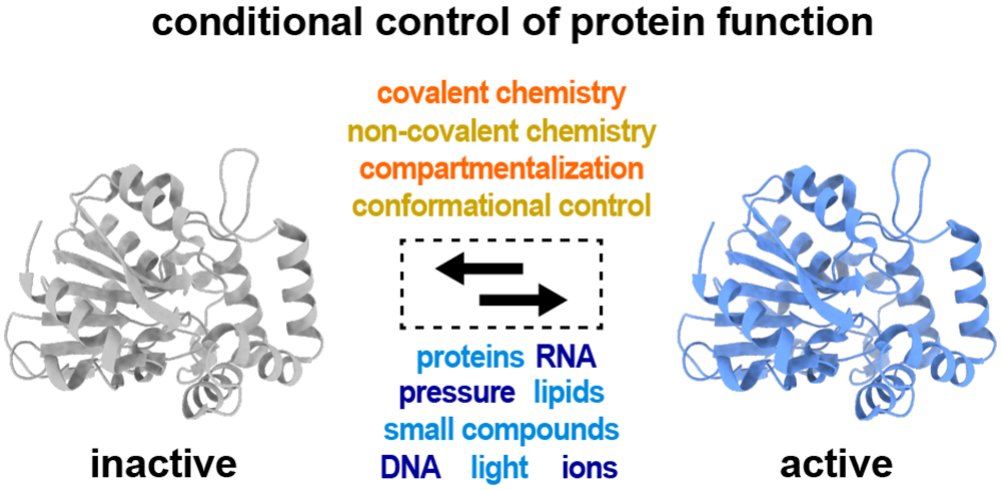

Design of the next-generation of therapeutics, biosensors, and molecular tools for basic research requires that we bring protein activity under control. Each protein has unique properties, and therefore, it is critical to tailor the current techniques to develop new regulatory methods and regulate new proteins of interest (POIs). This perspective gives an overview of the widely used stimuli and synthetic and natural methods for conditional regulation of proteins.

## Introduction

Protein regulation orchestrates nearly all molecular events of life. Its importance can be realized from the existence of about six million proteoforms which are produced from just ∼20000 human genes.^1,2^ This diversity of form and function is primarily achieved to control protein activity. A systematic control of protein function using various chemical and biochemical strategies is called protein regulation.^3^ Proteins are said to be conditionally regulated if these strategies are executed in response to a specific stimulus.

Biochemical reactions are largely performed in complex milieu rather than in isolation. Consequently, any participating species from reactions occurring in proximity can influence the reaction of interest. Therefore, life cannot persist if orthogonal mechanisms are not in place to regulate proteins in both the spatial and temporal dimensions. Understanding protein regulation is of utmost importance for chemists and biologists, and this review investigates the widely used techniques for protein regulation in biochemical research.

Several molecular techniques target protein activity at transcriptional or translational levels. While they have several advantages, they also have limitations and cannot be used to regulate all POIs, such as those required for viability.^4^ In contrast, chemical strategies allow the study of proteins that are otherwise intractable to genetic techniques, such as proteins that are essential in germline and are impossible to knock out or down with genetic manipulation. Chemical methods also achieve regulation at a faster rate as they affect protein activity at the post-translational level and cost less in comparison with tedious genetic engineering techniques.^5^ Some protein regulatory strategies also make use of a combination of chemical and molecular techniques, which are also discussed in this review.

**Figure 1:**
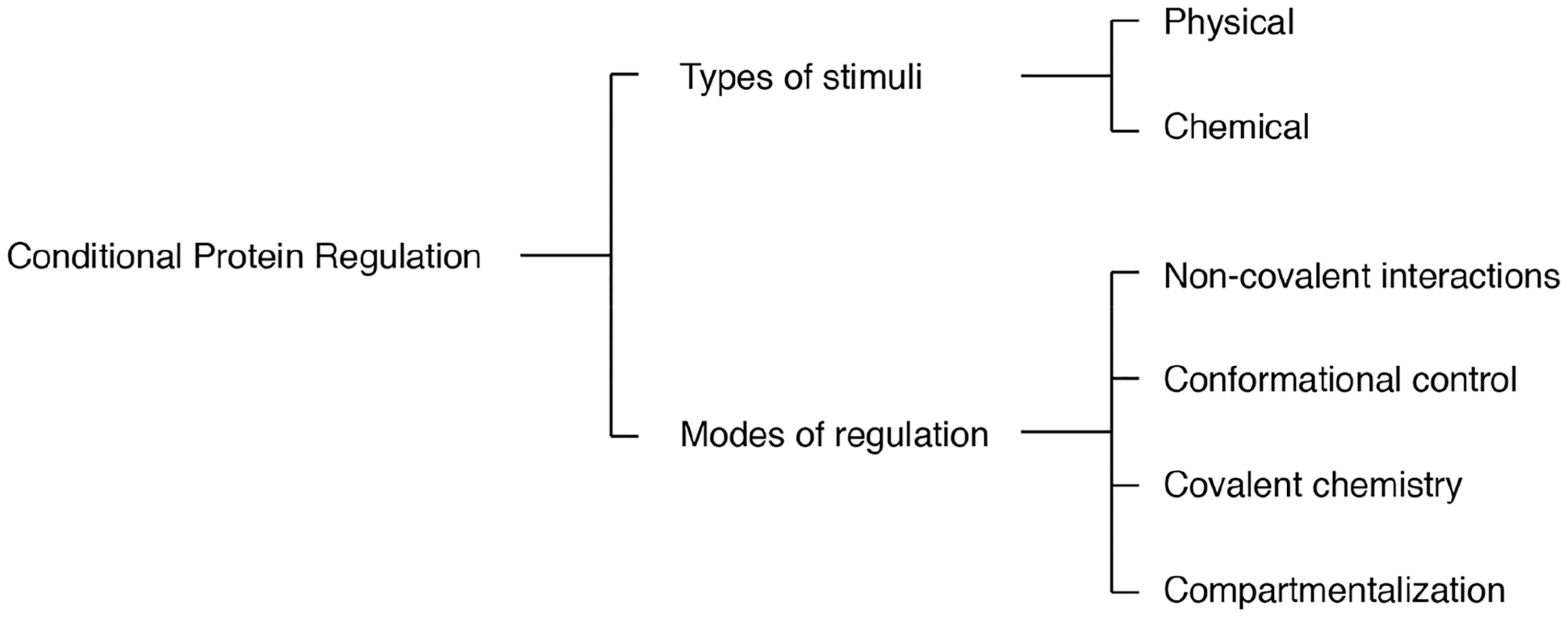
Outline of the modes of regulation and types of stimuli used for conditional regulation of proteins.

This perspective aims to provide an overview of different approaches acting at the post-translational level, rather than extensively reviewing each strategy. Conditional protein regulation can be systematically studied by understanding (a) the stimulus and (b) the method of regulation (Figure 1). Proteins are successfully regulated using specific combinations of stimuli and regulatory approaches, the most common of which are discussed in this review.

## Types of Stimuli

Broadly, both physical and chemical agents are known to regulate proteins. They generally induce changes in the protein by either making or breaking bonds, or changing its conformation or the nature of its interactions.

### Physical Stimuli

In nature, light is responsible for the regulation of photoreceptor proteins which participate in visual perception and phototaxis.^6,7^ By understanding the light-induced chemical and structural changes in such proteins which are discussed later in this perspective, several light-responsive moieties were developed to be incorporated into POIs to trigger structural and conformational changes on demand. Most light responsive elements in use respond to visible-NIR light as UV light (a) is toxic to nucleic acids and (b) cannot be used for *in vivo* applications due to low tissue penetration.^8^

Pressure, sound, and temperature are other physical stimuli for post-translational protein regulation.^9,10^ Both the pressure- and thermo-receptors belong to the same family of proteins, and they change their conformation to permit the movement of cations across cell membranes to convert mechanical forces into electrochemical signals. Sensing and responding to changes in temperature, blood pressure, touch etc. is typically accomplished by proteins responding to force.^11−14^ So far, temperature and pressure have been used less frequently for synthetic regulation of proteins.

### Chemical Stimuli

Chemical stimuli are responsible for the regulation of a significant proportion of proteins. Several chemicals ranging from ions to small- and biomolecules, which include lipids, DNA, RNA, and even other proteins, can act as stimuli.^15−17^ Small molecules act as stimuli at the transcriptional, translational, and post-translational levels, of which only those acting on proteins will be discussed in this text. Though biomolecules act as stimuli with higher specificity, they have short half-lives in plasma and cannot easily pass through membranes.^18^ Other noteworthy chemical stimuli include chemical gradients in the environment. For instance, pH gradients can be observed between different tissues or intracellular organelles which affect the protonation states and protein conformations and hence modulate their activity.^19−21^ Oxygen gradients affect proteins such as the hypoxia inducible factor, which is hydroxylated at prolines at high oxygen concentrations for degradation.^22−24^ Reactive oxygen species (ROS) act as stimuli for redox-switch proteins which may be regulated through chemistry at their reactive centers including thiols or metals.^25^ Synthetic hypoxia- and ROS-sensitive immolative moieties have also been developed for protein regulation.^26,27^ Finally, biomolecules themselves act as stimuli for protein regulation.^28^

## Modes of Regulation

A stimulus will guide the POI into either one of the paths leading to activation or inactivation. These paths or regulatory strategies can be studied by categorizing them as discussed in this text. Some proteins are also known to be regulated using a combination of these strategies.

### Regulating Activity through Noncovalent Interactions and Conformational Control

Many stimuli interact noncovalently with POIs by association or dissociation. Such interactions lead to changes in the protein interactome, conformations, and creation or destruction of enzymatic active sites to control biochemical activity.

**Figure 2:**
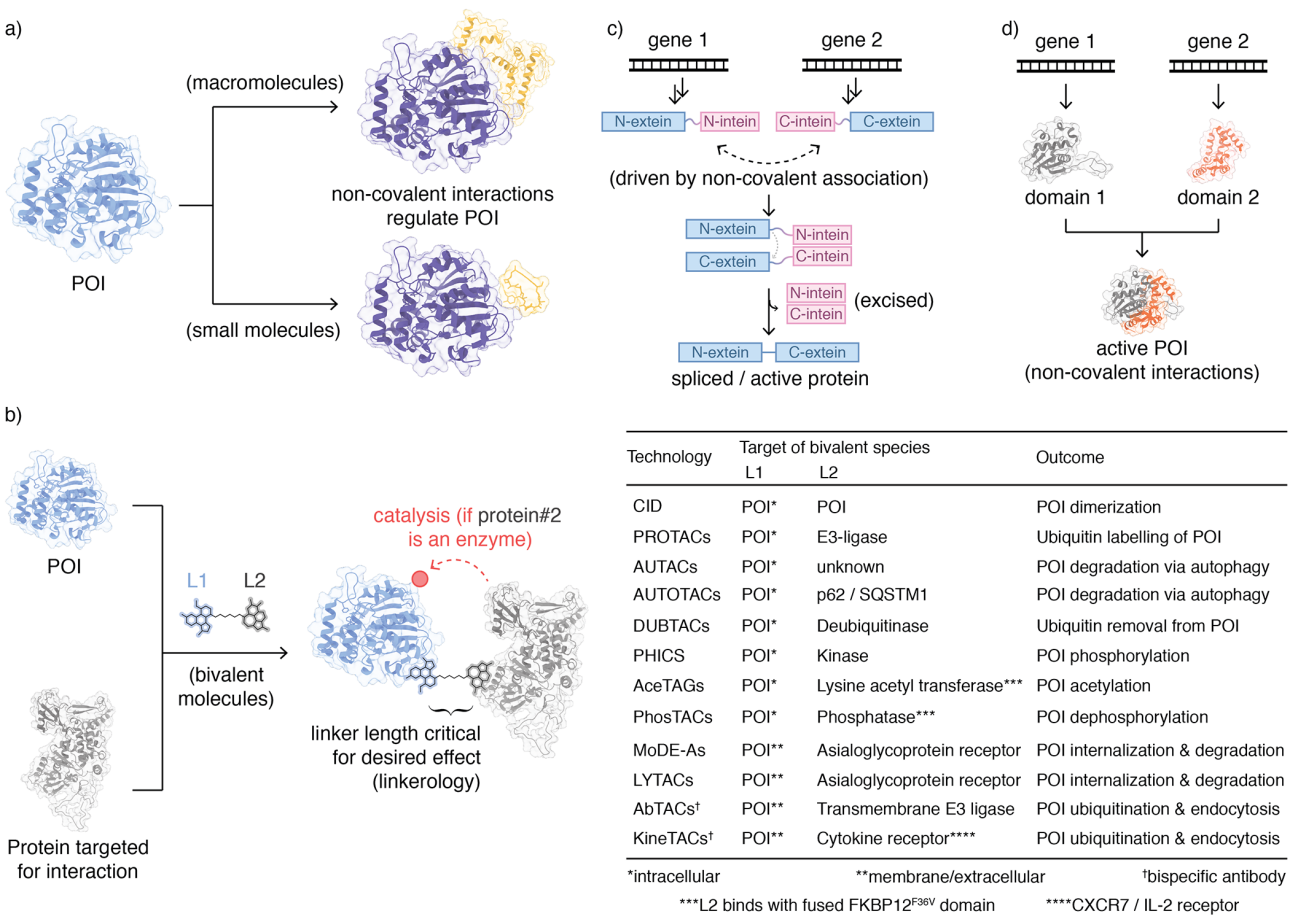
Regulation of protein activity through noncovalent interactions. (a) Small or macromolecules directly influence the activity of POI. (b) Different bivalent ligands designed to create neo interactions between two different POIs. (c) Split inteins. (d) Protein complementation.

The majority of pharmaceutical agents fall under this category (Figure 2a). It is beyond the scope of this review to discuss these examples in detail.^29−31^ Biologically, feedback inhibition of metabolic pathways occurs through noncovalent interactions.^32^ Sensor proteins, such as calmodulin and GTPases, are also regulated by conformational control. Additionally, it is important to note another type of allostery, viz., cooperativity which permits graded control of protein activity. Binding of oxygen at allosteric sites on hemoglobin is a well-studied example of cooperativity.^33^ The ability of small molecules to control protein complexation has been used in the pharmaceutical industry for therapeutic benefit and to control the delivery of biotherapeutics.^34−36^ If any POI is inert toward the action of a stimulus, a frequently employed strategy is to fuse the POI with a domain which recognizes a small molecule through noncovalent interactions.^37,38^

Many small molecules and drugs are also capable of inducing protein associations (Figure 2b).^39−42^ For instance, the immunosuppressant drug tacrolimus (FK-506) binds with a ubiquitously expressed 12 kDa protein called FKBP12.^43^ This interaction is very specific and therefore dimers of such compounds are used to induce protein interactions.^44−46^ Overall, this process was named chemically induced dimerization (CID), and was applied to study the effects of protein associations in living systems such as Ras signaling, Fas signaling, and T-cell receptor activation in lymphocytes, etc.^47,48^ CID did not just aid in making fundamental discoveries, but also was developed as a tool for the clinics to control the side effects of CAR T cell immunotherapy, and as a switch control in patients that develop Graft Versus Host Disease after T cell transplantation.^49−51^ CID is generally made possible with any POI by fusing it with binding domains genetically. Since the dimerizing molecule brings two fusion proteins together by binding with the fused domain, interactions of the POI can be studied without perturbing its chemistry or conformation. These bivalent ligands have been further modified to prevent their interactions with POIs until they encounter a specific stimulus. This additional layer of regulation made spatiotemporal control of CID possible by modifying the bivalent ligands to be sensitive to specific stimuli.^52−54^

Certain proteins such as the light-oxygen-voltage-sensing domains (LOV domains), chryptochromes (CRY2), and phytochromes (PHYB) change their conformations when exposed to light and undergo dimerization or oligomerization with their specific binding partners (light induced/light activated dimerization, LID/LAD).^55^ While these proteins naturally participate in cellular activities like phototropism and circadian rhythms, they are now extensively used by chemical biologists to study activities dependent on protein density. LOV and CRY2 domains require flavin cofactor (abundantly available in all cells) and respond to blue light. In contrast, PHYB requires the cofactor phycocyanobilin which is not available in animal systems. Therefore, phycocyanobilin must be included in those assay systems which use PHYB domains. The natural LOV domain comprises of a smaller cysteine containing “Per-Arnt-Sim” or PAS domain bound to the flavin cofactor. The cysteine in the PAS domain reacts with flavin covalently when exposed to light, leading to changes in conformation and unfolding of a specific α-helix in the LOV protein. The POI can be caged by fusing it with this α-helix and uncaged using light. This α-helix may also be engineered to bind with the POI and control protein–protein interactions. Furthermore, the LOV domain has been engineered to produce a variety of light-induced effects which have been reviewed extensively.^56^ Briefly, they can be used for controlling protein localization (optogenetic systems iLID and TULIP), producing homodimers (TAEL and Vivid) or heterodimers (Magnets). Light controlled dimerization (or multimerization) is also possible with another fluorescent protein, Dronpa (K145N mutant), which remains as a monomer in dark (∼500 nm). It associates into a tetramer when exposed to high energy light (∼400 nm). This property of the protein was utilized to control POIs. The association of a POI was controlled with light by expressing it as a fusion protein with with the Dronpa K145N domain on the N- or C-termini.^57,58^ Like CID, LAD using fusion proteins can be used to study protein associations without introducing conformational changes in the POI.

Obvious disadvantages for both CID and LAD must be noted. In addition to the use of genetic methods to incorporate these domains into the POI, fusion proteins do not accurately possess the properties of the natural POI. These domains are usually bulky and change the molecular weights, isoelectric points, and therefore, possibly the localization or interactions of the POI. Therefore, fusion proteins must be cautiously used only if the advantages of the study outweigh the disadvantages associated with the system.^59^

Other bi- or multivalent ligands are also designed to induce associations specifically between two or more POIs and *enzymes* (Figure 2b). The proximity of the two protein species increases their local concentration, thus forcing the POI to act as a substrate for the enzyme, altering its chemical nature. For instance, a well-designed proteolysis-targeting chimera (PROTAC) brings the POI closer in space with an E3-ligase. This leads to labeling of the POI with ubiquitin and the POI is consequently removed through proteasomal degradation.^60−63^ Molecular glues also aid in proteasomal degradation of POI(s); however, they are structurally different from PROTACs as they do not contain a linker connecting two independent ligands.^64^ They are single-unit entities with the capacity to establish new and stable interactions between the POI and the E3-ligase.^65−67^ Macroautophagy degradation targeting chimeras (MADTACs) including autophagy-targeting chimeras (AUTACs) and autophagosome-tethering compounds (ATTECs) utilize the mechanisms of autophagy to degrade the POI.^68^ The binder in the AUTACs is covalently linked with a guanylated cysteine, a moiety mimicking the protein post-translational modification S-guanylation. Proteins with S-guanylation are known to undergo degradation through autophagy via an unknown mechanism and AUTACs install this labeling specifically on the POI through directed and noncovalent interactions.^69^ AUTOTACs are a different class of bifunctional molecules that act intracellularly by binding with the POI on one terminus and p62 (SQSTM1) on the other. AUTOTAC induced association of the POI and p62 leads to the activation of autophagy pathways and degradation of the POI.^70^ ATTECs are originally molecular glues that interact with the POI and the autophagosome membrane protein LC3. The POI and LC3 interact through the interactions made by the ATTEC and are internalized into the autophagosome for degradation.^71^ Deubiquitinase-targeting chimeras (DUBTACs) mediate interactions between the POI and the deubiquitinase enzyme, which results in the removal of ubiquitin labels thereby preventing the degradation and increasing the half-life of the POI.^72^ Phosphorylation-inducing chimeric small molecules (PHICS) are bivalent molecules which bind with and bring together a kinase (either AMPK or PKC so far) and the POI to enable phosphoryl transfer on to the POI.^73^ Acetylation taggers (AceTAGs) and phosphorylation targeting chimeras (PhosTACs) do not directly bring the enzyme and POI together but make use of a FKBP12^F36V^ domain genetically installed on lysine acetyl transferase and Ser/Thr phosphatase enzymes respectively to mediate any associations between them.^74,75^

Transmembrane proteins, which include ion channels, molecular receptors, adhesins etc., constitute a significant proportion of the functional proteome and can be important targets in drug discovery. Conditional control of transmembrane POIs thus enables the development of new therapies. However, design of bifunctional molecules for this purpose is a challenging task as they generally are incapable of bringing the transmembrane POI and any cytosolic enzyme closer in space as the majority of the POI is embedded in lipid layers, thus making it less accessible. Strategies which have been successful so far target these POIs on their extracellular domains. Molecular degraders of extracellular proteins through the asialoglycoprotein receptor (MoDE-As) and lysosome-targeting chimeras (LYTACs) are generally mannoslyated and bring the membrane POI and mannose-6-phosphate (M6P) receptor together, whose association is followed by internalization of the receptor-POI complex into a lysosome for degradation.^76,77^ In addition to transmembrane POIs, both these bivalent ligands are capable of targeting extracellular proteins for degradation via interactions with the M6P receptor, followed by lysosomal degradation. Antibody-based PROTACs (AbTACs) and Proteolysis Targeting Antibodies (PROTABs) are bispecific IgG antibodies which have the arms engineered to bind with (a) a POI and (b) a transmembrane E3 ligase like RNF43 or ZNRF3.^78,79^ Both PROTABs and AbTACs were observed to ubiquitinate the POI resulting in its endocytosis and/or degradation. GlueTACs are engineered nanobodies consisting of (a) a proximity reactive noncanonical amino acid on its paratope and (b) a cell-penetrating peptide and lysosomal sorting sequence (CPP-LSS) away from the binding site. GlueTACs covalently label the membrane POI using the noncanonical residue, and are internalized into the lysosome for degradation due to the presence of the CPP-LSS moiety.^80^ Cytokine receptor-targeting chimeras (KineTACs) are engineered antibodies, like PROTABs, that also have two arms which bind with the POI (extracellular/membrane) and the cytokine receptor, respectively. Upon association, the POI-KineTAC-cytokine receptor complex is internalized into lysosomes for degradation.^81^

Unlike the small molecules discussed above which aid in the association of proteins, protein–protein interaction modulators (PPI modulators) prevent such protein–protein interactions (PPIs) by binding noncovalently at either of the protein interfaces aiding in their dissociation. The interfaces involved in the interactions are generally large in comparison with the active or allosteric binding site of a small molecule on a protein or an enzyme. Moreover, these interfaces are flat and the majority contain hydrophobic residues thus making an efficient PPI modulator design very difficult.^82,83^

Split inteins are typical examples of protein activation upon association (Figure 2c). The POI is generally expressed as two different N-terminal and C-terminal segments, each containing two half-domains of N- and C- exteins and inteins, respectively. Protein splicing, i.e. covalent chemistry is initiated only when the two N- and C-terminal domains are brought together by noncovalent interactions. Splicing of the protein occurs through a series of additions/eliminations, ultimately resulting in the generation of the POI. Inteins occur in nature in all domains of life but also have been recently used as chemical biology tools, including in living cells.^84,85^

Complementation is another strategy typically requiring noncovalent associations for protein activation (Figure 2d). In this approach, the POI is split into two domains such that they are inactive. Biological activity of the POI is restored by the interaction between the two domains. Complementation is widely used in molecular biology, generally applied toward enzymes to be used as reporters in specific assays (split protein assays). Besides using this approach for basic research, it is also applied for tissue-targeted activation of therapeutic proteins.^86,87^ Complementation has also been applied to improve the therapeutic potential of recombinant cytokines, the use of which is limited in the clinics due to severe side effects. A two-component strategy was developed that requires localization that can be independently targeted to restrict activity to cells expressing two surface markers.^88^ The association of the two components regenerates Neoleukin-2/15, a IL-2/15 mimetic designed for both *trans*-activating immune cells surrounding the target cancer cells and *cis*-activating to directly target immune cells.^89^ This technique is also successfully used for mitochondrial genome editing, specifically deamination of cytidines in dsDNA using DddA-derived cytosine base editor (DdCBE). This protein is an interbacterial toxin which potentially has toxicity. To lower this toxicity and off target reactions, DdCBE is engineered into split inactive protein domains. Cytidine deamination is initiated only by the fusion of the two domains near the target DNA.^90^ Nanobody-fused split O-GlcNAcase (nano-OGA - also discussed later) are enzymes that remove the *O*-GlcNAc post-translational modification from the POI. This protein is engineered into split domains to reduce its off-target activity, thus using complementation to improve the selectivity of the enzyme toward the POI.^91^ Complementation also is shown to be effective for improving proteomics studies. Split-TurboID cannot biotinylate proteins until the domains associate in the presence of rapamycin (CID). The resolution of mass spectrometry-based proteomics when TurboID complementation was achieved within specific organelles was improved, resulting in the identification of new proteins.^92^

**Figure 3:**
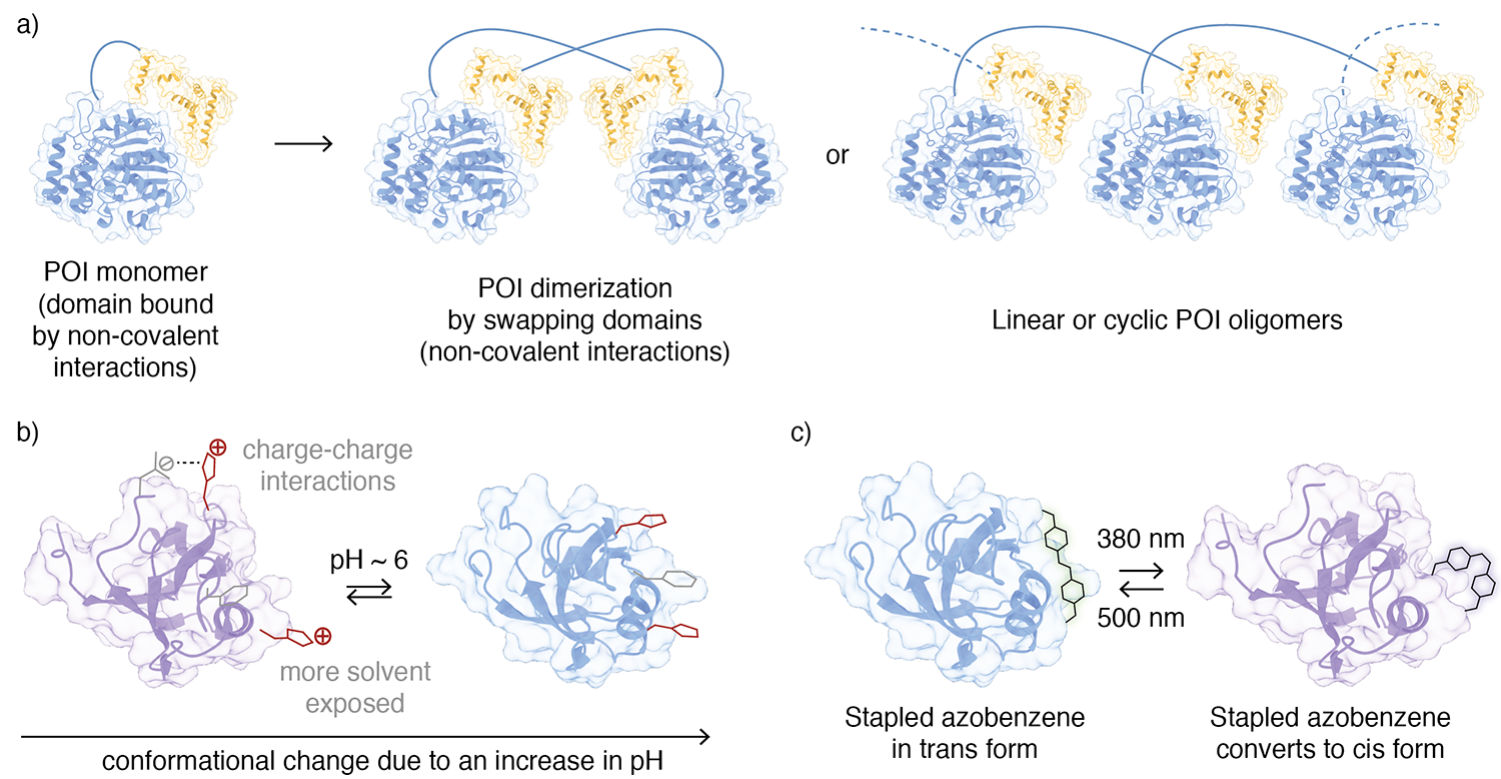
Protein regulation through (a) domain swapping, (b) histidine switches, and (c) azobenzenes.

Domain swapping is generally applied to produce dimers or oligomers of a POI (Figure 3a). Here, the POI has a domain bound to itself, whose interactions can be substituted with those of another domain from the neighboring POI.^93^ This leads to dimerization; other possibilities of substitutions (i.e., domain swapping between several molecules of POI) are also possible leading to the formation of oligomers and sometimes, protein fibrils.^94^ This technique has been synthetically applied to POIs to regulate their activities under the influence of specific stimuli.^95,96^

Design of protein conformational switches is a challenging task as proteins tend to change their conformations with minute changes in the environment.^97,98^ However, a few methods have been developed for specific proteins; but they may not be applied to any POI. Histidine residues are well studied and used for achieving pH responsive conformational control by incorporation at specific sites in proteins; their participation in protein folding through intramolecular hydrogen bonds can be controlled with pH as their protonation at any pH below ∼6.5 will result in the loss of hydrogen bonds, ultimately leading to large changes in protein conformation (Figure 3b).^99−102^ While drastic changes in temperature result in irreversible conformational changes in any protein, reversible temperature responsive conformational switches have also been identified. This type of switching sometimes results in the gel–sol phase transition of peptides and is therefore extensively explored by material scientists for making temperature-responsive hydrogels.^103,104^ Azobenzenes and their next generation derivatives are either cross-linked over two residues or incorporated directly into the backbone of a POI, which when exposed to specific wavelengths of light undergo cis–trans isomerization and a subsequent conformational change (Figure 3c).^105,106^

### Regulating Activity with Covalent Connections

A significant proportion of proteins are regulated through covalent chemistry. Any covalent modification on a protein will lead to changes in local charges/interactions or conformations as discussed in the previous sections.

One of the most widely employed ways to regulate proteins *in nature* is using enzyme catalyzed post-translational modifications (PTMs). Kinases and phosphatases catalyze the addition and removal of phosphate groups respectively from their target proteins. This process generally occurs on serine, threonine, and tyrosine residues on the POI. This type of regulation is so widely used that about 3% of the proteins in yeast are kinases and phosphatases. It must also be highlighted that this method of regulation is chosen to control the activity of numerous classes of proteins including ion channels, membrane and structural proteins, enzymes etc.^33^ Several PTMs like ubiquitination, performed by ubiquitinases and deubiquitinases, are targeted toward lysine residues on different proteins.^107^ Besides labeling the protein for degradation, ubiquitination is also used as a strategy to control protein localization and interactions.^108^ Additionally, the post-translational covalent modifications on histones too are greatly studied to understand epigenetics and gene expression.^109^

**Figure 4:**
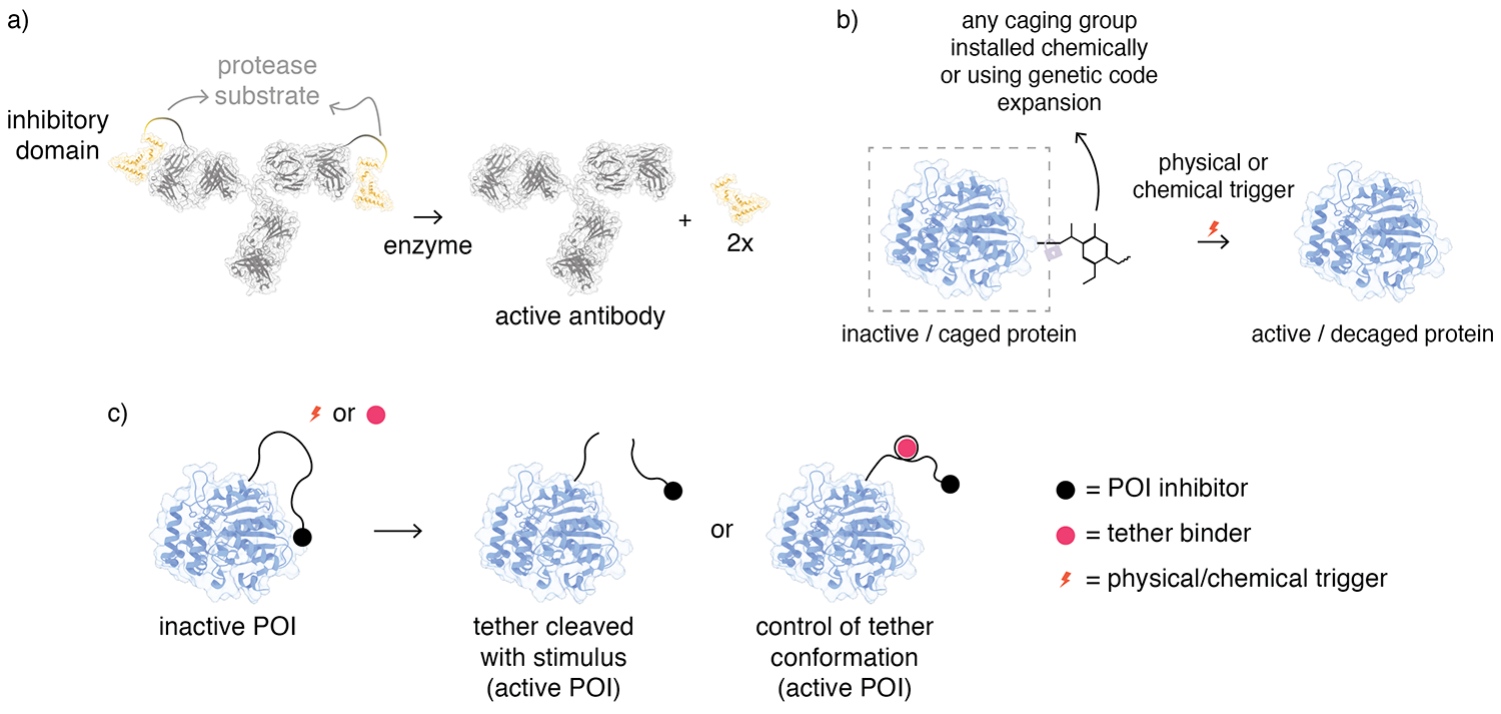
Protein regulation via (a) proteolytic action, (b) caging/decaging chemistry, and (c) tethered pharmacology.

Proteolytic action is an *irreversible* approach for protein regulation. Proteolysis itself can also be regulated by other proteases; the pancreas produces inactivated enzymes called zymogens, which are activated in the small intestine by other specific proteases. Most protein hormones are first created as pro-hormones and are activated into hormones by proteolytic activity.^110^ Viruses also express pro-proteins, which must be hydrolyzed by proteases to form functional proteins as in the case of HIV.^111^ Critical processes such as blood clotting occur only through the proteolytic activation of the coagulation cascade.^112^ One of the applications of this irreversible covalent chemistry is the development of enzyme-activable therapeutics. Bulky moieties are introduced on the POI to preserve it in an inert state until it encounters an enzyme that cleaves off these moieties to activate the POI.^113−117^ Antibodies are also regulated in a similar fashion by introducing cleavable bulky moieties on the paratope (Figure 4a).^118−120^ On the other hand, certain proteins or peptides are required to be protected from proteolytic activity. Peptide macrocyclization and site-specific conjugation (PEGylation, etc.) are strategies which are widely used for this purpose. Cyclization restricts peptides from attaining a specific conformation for recognition by proteases, and PEGylation prevents any POI-protease interactions.^121,122^

Many small molecules react covalently with proteins at allosteric sites.^123^ These covalent allosteric inhibitors are generally irreversible; they trap the POI in a specific conformation for a long duration via a two-step process. The first event is relatively fast and involves the binding of the small molecule at the allosteric site, followed by a second slow event which involves the covalent modification of the target protein.^124^ Any allosteric interaction ultimately alters protein activity by inducing conformational changes. Design of covalent allosteric inhibitors as drugs is a challenging task and they can cross-react with other homologous proteins resulting in adverse effects. On the other hand, it is observed that drug-resistance is not acquired with covalent allosteric inhibitors.^125^

A significant proportion of covalent inhibitors are directed toward the active site of an enzyme. The small molecules have other chemical groups such as hydrogen bond donors/acceptors in addition to the reactive functionality to increase their specificity toward a particular enzyme. Suicide inhibitors also bind at the active sites of the enzyme and make covalent connections during the catalysis, thus rendering the enzyme inactive. Both these types of active site-directed covalent inhibitors affect the activity of an enzyme by preventing the entry and catalysis of its substrate.^126−128^

Chemical cross-linking is another strategy to regulate proteins. This is achieved through bioconjugate chemistry, or bioorthogonal reactions or enzymatic labeling as in the case of protein–protein (homo- or hetero-) dimers.^129−132^ Alternatively, bifunctional molecules like glutaraldehyde or bis-acids (EDC/NHS chemistry) may be used to cross-link proteins on their basic residues like lysine nonspecifically.^133,134^ The latter approach, though very efficient, may ultimately inactivate the protein. In the context of vaccine design, glutaraldehyde-based cross-linking was used to stabilize the HIV envelope glycoprotein BG505 to improve neutralizing antibodies against HIV.^135^

Unlike the split inteins discussed earlier, inteins are fully hosted by the protein from which they autocatalytically excise themselves. The chemistry of excision is the same as seen in split inteins. Protein splicing has predominately been utilized in the areas of protein purification and protein cyclization.^136^ However, by combining an intein domain with sensing and reporter domains, the rate of protein splicing activity can be controlled by external stimuli leading to the emergence of conditional protein splicing (CPS).^137,138^ Numerous examples of CPS have been reported with small molecules, light, temperature, and pH.^139,140^ Poor solubility, local environment optimization and limited reversibility are typical problems in the development of CPS as a generic method.

Protein activity can be temporarily turned off by caging the POI using site- or ligand-specific chemistry (Figure 4b). The enzymatic active site or a binding interface of a POI are the target sites for installation of caging groups. Decaging occurs when these functional groups are exposed to specific physical or chemical stimuli including small molecules, light, metal ions, or enzymes.^141−148^ Unnatural amino acids (UAAs) present bioorthogonal functionalities for easy and site-specific incorporation of caging groups. Alternatively, caged residues can be directly introduced at desired sites in the POI using genetic code expansion (GCE) and specific stimuli can be used to decage these residues on demand.^149−153^ UAAs were also introduced in proteins to permit cleavage of the peptide backbone at specific sites with an appropriate stimulus.^154^ A recent advancement in GCE for conditional protein regulation is CAGE-prox (computationally aided and genetically encoded proximal decaging strategy). This approach relies on computational tools to identify a key residue in proximity to the active site, which can be replaced with a caged residue to conditionally prevent substrates from entering the active site.^155^ GCE also allows incorporation of diazirine and tetrazole based UAAs (cysteine, selenocysteine, or lysine) for photo-cross-linking to study protein–protein interactions. These proteins can then be unlinked using hydrogen peroxide.^156−158^

In tethered pharmacology, proteins are regulated by conjugating a tether to the POI which generally terminates with a small- or biomolecule that binds weakly with the POI, thereby inhibiting its activity (Figure 4c). Tethering lowers the translational entropy, thereby increasing the affinity of the binder for the POI. In the presence of a stimulus, the tether can be either cleaved covalently or altered conformationally to conditionally unbind the small-/biomolecule from the POI, thus activating it.^159−161^ Enzymes have been successfully regulated using this strategy by tethering them with an active- or allosteric-site binder.^162−164^ Both natural and unnatural polymers have been used to construct the tethers.

### Regulating Activity by Compartmentalization

**Figure 5:**
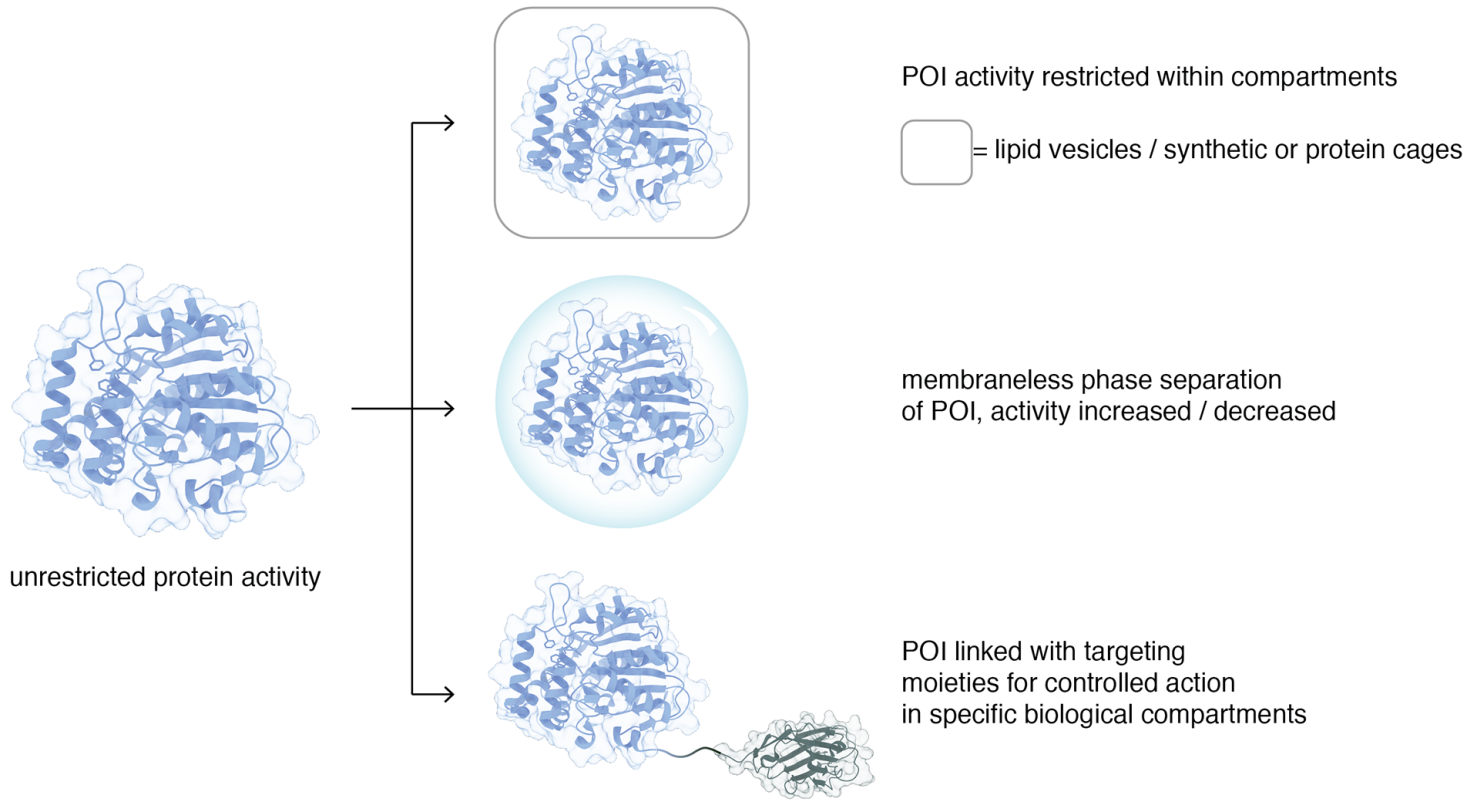
Protein regulation by compartmentalization.

In this approach, the POI is generally not modified or influenced by an effector to modulate its conformation. Activity is only controlled by removing or concentrating the POI at the site of action. The compartment may or may not be a physical enclosure. Such approaches are discussed below (Figure 5).

Protein activity may be directed toward specific sites without the use of physical enclosures such as membranes. This is typically achieved by linking the POI with antibodies or lectins that bind with specific biomolecules expressed exclusively by the respective compartments in the body.^165^ Antibody-protein conjugates or fusion proteins are generally used for extracellular tissue targeted delivery. Comparatively, intracellular targeting of proteins is further challenging as it requires translocation across (multiple) biological membranes. While a few strategies have been reported, there is still need for a robust generic method for delivering intact proteins across membranes.^166−168^ Nano-OGT/OGA (nanobody fused O-GlcNAc transferase/O-GlcNAcase) are enzyme-nanobody constructs which can be used to install/remove O-GlcNAc modifications on selective proteins. The nanobody helps direct the enzyme toward the POI, thereby increasing the effective concentrations of the enzyme and POI for improved biocatalysis.^91,169^

Alternative chemical methods also permit localization of proteins at desired sites without a physical enclosure. Lipidation is a popular approach in which the POI is covalently attached with lipid anchors to direct a protein to particular membrane sites. One prominent chemical lipidation approach uses a photoinduced tetrazole-alkene cycloaddition reaction to install exogenous lipid dipolarophiles onto proteins in live cells.^170^ Amphiphile-mediated depalmitoylation, on the other hand, aids in the removal of lipid modifications from proteins. Specifically, a cysteine containing amphiphile is observed to react with the thioester linkage of a S-palmitoylated membrane bound protein, thus unbinding the POI from the membrane via a native chemical ligation-like reaction.^171^ GCE (discussed earlier) was also applied to control post-translational modification of RanGAP1, the POI in this study and its localization at the nuclear membrane. Incorporation of a lysine protected with an aryl-azido group (PABK) prevented SUMOylation of the POI. Decaging the lysine with 2-(diphenylphosphanyl)benzamide localized the POI at the nuclear membrane through interactions with the nuclear pore complex.^172^ Magnetic control of proteins can also be achieved by conjugating or fusing the POI with an antibody-functionalized with superparamagnetic nanoparticles.^173^ Alternatively, a functionalized antibody can be directly used to bring together the POI in the cell through noncovalent interactions, whose localization can be controlled using an electromagnetic needle.^174^ The anchor away system is another technique used to evacuate nuclear POIs conditionally and rapidly away from the nucleus using specific chemical stimuli to tether them in the cytoplasm resulting in their loss of function. This loss of function is reversible and can be restored when the chemical stimulus is removed. Multiple nuclear proteins can be regulated simultaneously using this system.^175,176^ The PhyB and LOV2 systems, described under light induced dimerization, have also been used to localize proteins intracellularly with blue or NIR light.^177^

Encapsulation of POIs in membranes or cages is an effective way of spatially controlling protein activity. POIs have been encapsulated by complex coacervation with poly ionic peptides or compounds to improve their stability in circulation.^178,179^ Although few studies show that proteins lose their integrity when encapsulated in lipid membranes, some have encapsulated peptide- and protein-therapeutics to explore their capacity for delivery.^180−183^ Furthermore, these membranes are functionalized for targeted therapy or membrane permeation.^184,185^ Both synthetic and protein-based cages have been used to encapsulate POIs.^186,187^

Some proteins are also regulated by liquid–liquid phase separation (LLPS), initiated by both chemical and physical stimuli.^188,189^ LLPS can either lead to reduction in protein activity as it is removed from the reaction mixture or improvement of the same due to increased concentration within the excluded compartment. One of the major requirements for a protein undergoing LLPS is the presence of a charged intrinsically disordered domain (IDD), which provides the POI a high conformational entropy and freedom to explore a variety of conformations.^190,191^ IDDs generally contain many residues. While peptides containing 6–18 residues were observed to induce phase separation when incorporated at the C-termini of specific POIs, short peptides generally cannot induce LLPS for any POI as they do not contain the structural features discussed above.^192^ Both experimental and computational attempts are being made to predict the structure–LLPS relationships of peptides.^193−197^ Synthetic protein-recruiting/-releasing condensates (SPRECs) are designed which are capable of sequestering POIs, as well as releasing them in living systems. Reversible POI sequestering was achieved by controlling its associations with the phase separating protein using the LOV systems discussed previously for light induced association and dissociation, respectively.^198^ Molecular biology techniques are currently being used to construct fusion proteins of any POI with an IDD to allow controlled phase separation. Stimuli such as light and small molecules are being explored for conditional regulation.^199−201^ Though LLPS is a powerful tool for protein regulation, it should be noted that this process can initiate protein aggregation and misfolding, thus leading to many problems. A successful strategy for conditional regulation must be reversible and maintain protein quality after LLPS.

## Conclusions

Protein regulation is not just central to life, but also to the majority of fundamental discoveries, preclinical research and therapeutic design. With rapid advancement in the fields of protein engineering and bioconjugation, new proteins are constantly developed which require intelligent methods of regulation. The recent discovery of a NOS bridge in proteins as a redox switch is another reminder that the field of protein regulation is vastly unexplored.^202,203^ Preclinical studies have repeatedly shown that conditionally regulated proteins are superior to their native forms. However, most biotechnological products in clinics are not conditionally regulated; instead, native protein forms are used as therapeutics. There is a need for the development of robust methods such that they will be translated into clinical settings and help the development of safer and improved biotherapeutics.

Biological methods are not cost-effective; however they are generic and reliable. For instance, small and easy design modifications to respective plasmids can allow knock of any DNA sequence using CRISPR. Therefore, they are widely used by both chemists and biologists. So far, very few generic chemical tools have been developed, which too are difficult to be translated for regulating other POIs. Moreover, the majority of the techniques require the use of biological procedures like protein engineering or expression as discussed in this perspective. We believe that it is for this reason chemical approaches (at a post-translational level) are not appreciated enough for translational research yet.

Biological tools have also become successful because of the utilization of the host’s biosynthetic machinery to obtain the desired effect. *In situ* biosynthesis of large proteins and nucleic acids, which are extremely crucial for biocatalysis and molecular recognition, makes the development of biological tools easier in comparison. On the other hand, chemical methods must overcome the challenges in both the synthesis and delivery of such complex biochemical components to the target site. This limits the rate of development of chemical approaches for conditional protein regulation.

The goal of a chemical regulatory method is to achieve the desired biological effect on a specific POI. This can be achieved using site- and residue-specific chemistry which is bioorthogonal to any other processes using water-soluble and readily activated reagents. Therefore, the growth of this technology, i.e., conditional protein regulation, also depends on how fast the progress is in the field of bioconjugation. Chemists are now providing elegant and inexpensive solutions to this problem. To overcome the problems of molecular recognition and reactivity for instance, they are making use of residue specific reagents in combination with targeting moieties such as antibodies or ligands identified via high throughput screening and mass spectrometry. We anticipate that more chemical methods will be developed in the coming years with the expansion of our bioconjugate chemistry toolbox. In addition, there is vast space for the evolution of new chemistry with the discovery of novel biochemical regulatory processes. Such new methods of regulation may broadly fall under one of the categories discussed in this review. We hope that this perspective serves as a guide for those within and outside of the community and inspires the next generation of researchers to take up these challenges.

## References

[ref1] Ponomarenko E. A.; Poverennaya E. V.; Ilgisonis E. V.; Pyatnitskiy M. A.; Kopylov A. T.; Zgoda V. G.; Lisitsa A. V.; Archakov A. I. The Size of the Human Proteome: The Width and Depth. Int. J. Anal. Chem. 2016, 2016, 1–6. 10.1155/2016/7436849. PMC4889822 27298622

[ref2] Omenn G. S.; Lane L.; Overall C. M.; Cristea I. M.; Corrales F. J.; Lindskog C.; Paik Y.-K.; Van Eyk J. E.; Liu S.; Pennington S. R.; others; et al. Research on the Human Proteome Reaches a Major Milestone:> 90% of Predicted Human Proteins Now Credibly Detected, According to the HUPO Human Proteome Project. J. Proteome Res. 2020, 19 (12), 4735–4746. 10.1021/acs.jproteome.0c00485. 32931287 PMC7718309

[ref3] Lodish H. F.; Berk A.; Kaiser C. A.; Krieger M.; Bretscher A.; Ploegh H.; Amon A.; Martin K. C. Molecular Cell Biology, 8th ed.; W.H. Freeman and Company: New York, 2016.

[ref4] Thomas L. W.; Esposito C.; Morgan R. E.; Price S.; Young J.; Williams S. P.; Maddalena L. A.; McDermott U.; Ashcroft M. Genome-Wide CRISPR/Cas9 Deletion Screen Defines Mitochondrial Gene Essentiality and Identifies Routes for Tumour Cell Viability in Hypoxia. Commun. Biol. 2021, 4 (1), 615 10.1038/s42003-021-02098-x. 34021238 PMC8140129

[ref5] Nadendla K.; Sarode B.; Friedman S. H. Chemical Modification of Proteins with Photocleavable Groups. In Methods in Enzymology; Elsevier, 2019; Vol. 624, pp 113–128. 10.1016/bs.mie.2019.04.008. 31370926 PMC7050930

[ref6] Pugh E. N.; Nikonov S.; Lamb T. D. Molecular Mechanisms of Vertebrate Photoreceptor Light Adaptation. Curr. Opin. Neurobiol. 1999, 9 (4), 410–418. 10.1016/S0959-4388(99)80062-2. 10448166

[ref7] Foster K. W.; Saranak J.; Patel N.; Zarilli G.; Okabe M.; Kline T.; Nakanishi K. A Rhodopsin Is the Functional Photoreceptor for Phototaxis in the Unicellular Eukaryote Chlamydomonas. Nature 1984, 311 (5988), 756–759. 10.1038/311756a0. 6493336

[ref8] Kielbassa C. Wavelength Dependence of Oxidative DNA Damage Induced by UV and Visible Light. Carcinogenesis 1997, 18 (4), 811–816. 10.1093/carcin/18.4.811. 9111219

[ref9] Coste B.; Mathur J.; Schmidt M.; Earley T. J.; Ranade S.; Petrus M. J.; Dubin A. E.; Patapoutian A. Piezo1 and Piezo2 Are Essential Components of Distinct Mechanically Activated Cation Channels. Science 2010, 330 (6000), 55–60. 10.1126/science.1193270. 20813920 PMC3062430

[ref10] Piraner D. I.; Farhadi A.; Davis H. C.; Wu D.; Maresca D.; Szablowski J. O.; Shapiro M. G. Going Deeper: Biomolecular Tools for Acoustic and Magnetic Imaging and Control of Cellular Function. Biochemistry 2017, 56 (39), 5202–5209. 10.1021/acs.biochem.7b00443. 28782927 PMC6058970

[ref11] Beech D. J.; Kalli A. C. Force Sensing by Piezo Channels in Cardiovascular Health and Disease. Arterioscler. Thromb. Vasc. Biol. 2019, 39 (11), 2228–2239. 10.1161/ATVBAHA.119.313348. 31533470 PMC6818984

[ref12] Wu J.; Lewis A. H.; Grandl J. Touch, Tension, and Transduction – The Function and Regulation of Piezo Ion Channels. Trends Biochem. Sci. 2017, 42 (1), 57–71. 10.1016/j.tibs.2016.09.004. 27743844 PMC5407468

[ref13] Moran M. M.; Xu H.; Clapham D. E. TRP Ion Channels in the Nervous System. Curr. Opin. Neurobiol. 2004, 14 (3), 362–369. 10.1016/j.conb.2004.05.003. 15194117

[ref14] Mundt N.; Spehr M.; Lishko P. V. TRPV4 Is the Temperature-Sensitive Ion Channel of Human Sperm. eLife 2018, 7, e35853 10.7554/eLife.35853. 29963982 PMC6051745

[ref15] Newton A. C. Lipid Activation of Protein Kinases. J. Lipid Res. 2009, 50, S266–S271. 10.1194/jlr.R800064-JLR200. 19033211 PMC2674703

[ref16] Liang S.; Blundell T. L. Human DNA-Dependent Protein Kinase Activation Mechanism. Nat. Struct. Mol. Biol. 2023, 10.1038/s41594-022-00881-w. PMC9935390 36604499

[ref17] McKenna S. A.; Lindhout D. A.; Kim I.; Liu C. W.; Gelev V. M.; Wagner G.; Puglisi J. D. Molecular Framework for the Activation of RNA-Dependent Protein Kinase. J. Biol. Chem. 2007, 282 (15), 11474–11486. 10.1074/jbc.M700301200. 17284445

[ref18] Yang N. J.; Hinner M. J. Getting Across the Cell Membrane: An Overview for Small Molecules, Peptides, and Proteins. In Site-Specific Protein Labeling; Gautier A., Hinner M. J., Eds.; Methods in Molecular Biology; Springer: New York, NY, 2015; Vol. 1266, pp 29–53. 10.1007/978-1-4939-2272-7_3. PMC4891184 25560066

[ref19] Casey J. R.; Grinstein S.; Orlowski J. Sensors and Regulators of Intracellular PH. Nat. Rev. Mol. Cell Biol. 2010, 11 (1), 50–61. 10.1038/nrm2820. 19997129

[ref20] Martin C.; Pedersen S. F.; Schwab A.; Stock C. Intracellular PH Gradients in Migrating Cells. Am. J. Physiol.-Cell Physiol. 2011, 300 (3), C490–C495. 10.1152/ajpcell.00280.2010. 21148407

[ref21] Andreeva D. V.; Melnyk I.; Baidukova O.; Skorb E. V. Local PH Gradient Initiated by Light on TiO _2_ for Light-Triggered Modulation of Polyhistidine-Tagged Proteins. ChemElectroChem. 2016, 3 (9), 1306–1310. 10.1002/celc.201600268.

[ref22] Safran M.; Kaelin W. G. HIF Hydroxylation and the Mammalian Oxygen-Sensing Pathway. J. Clin. Invest. 2003, 111 (6), 779–783. 10.1172/JCI200318181. 12639980 PMC153778

[ref23] Semenza G. L. Regulation of Mammalian O _2_ Homeostasis by Hypoxia-Inducible Factor 1. Annu. Rev. Cell Dev. Biol. 1999, 15 (1), 551–578. 10.1146/annurev.cellbio.15.1.551. 10611972

[ref24] Pugh C. W.; Ratcliffe P. J. Regulation of Angiogenesis by Hypoxia: Role of the HIF System. Nat. Med. 2003, 9 (6), 677–684. 10.1038/nm0603-677. 12778166

[ref25] Dickinson B. C.; Chang C. J. Chemistry and Biology of Reactive Oxygen Species in Signaling or Stress Responses. Nat. Chem. Biol. 2011, 7 (8), 504–511. 10.1038/nchembio.607. 21769097 PMC3390228

[ref26] Leriche G.; Chisholm L.; Wagner A. Cleavable Linkers in Chemical Biology. Bioorg. Med. Chem. 2012, 20 (2), 571–582. 10.1016/j.bmc.2011.07.048. 21880494

[ref27] Wang Y.; Xiao D.; Li J.; Fan S.; Xie F.; Zhong W.; Zhou X.; Li S. From Prodrug to Pro-Prodrug: Hypoxia-Sensitive Antibody–Drug Conjugates. Signal Transduct. Target. Ther. 2022, 7 (1), 20 10.1038/s41392-021-00833-8. 35058439 PMC8776858

[ref28] Chaires J. B. Allostery: DNA Does It, Too. ACS Chem. Biol. 2008, 3 (4), 207–209. 10.1021/cb800070s. 18422302

[ref29] Protein Allostery in Drug Discovery. Advances in Experimental Medicine and Biology; Zhang J., Nussinov R., Eds.; Springer: Singapore, 2019.

[ref30] Robertson J. G. Mechanistic Basis of Enzyme-Targeted Drugs. Biochemistry 2005, 44 (15), 5561–5571. 10.1021/bi050247e. 15823014

[ref31] Sunke R.; Adepu R.; Kapavarapu R.; Chintala S.; Meda C. L. T.; Parsa K. V. L.; Pal M. Vinylic Amino Group Activation: A New and General Strategy Leading to Functionalized Fused Heteroaromatics. Chem. Commun. 2013, 49 (34), 3570 10.1039/c3cc41337c. 23525274

[ref32] Ferrier D. R. Biochemistry, 6th ed.; Lippincott’s Illustrated Reviews; Wolters Kluwer Health/Lippincott Williams & Wilkins: Philadelphia, 2014.

[ref33] Molecular Cell Biology, 6th ed.; Lodish H. F., Ed.; W.H. Freeman: New York, 2008.

[ref34] Jonassen I.; Havelund S.; Hoeg-Jensen T.; Steensgaard D. B.; Wahlund P.-O.; Ribel U. Design of the Novel Protraction Mechanism of Insulin Degludec, an Ultra-Long-Acting Basal Insulin. Pharm. Res. 2012, 29 (8), 2104–2114. 10.1007/s11095-012-0739-z. 22485010 PMC3399081

[ref35] Löhr T.; Kohlhoff K.; Heller G. T.; Camilloni C.; Vendruscolo M. A Small Molecule Stabilizes the Disordered Native State of the Alzheimer’s Aβ Peptide. ACS Chem. Neurosci. 2022, 13 (12), 1738–1745. 10.1021/acschemneuro.2c00116. 35649268 PMC9204762

[ref36] Dunn M. F. Zinc–Ligand Interactions Modulate Assembly and Stability of the Insulin Hexamer – A Review. BioMetals 2005, 18 (4), 295–303. 10.1007/s10534-005-3685-y. 16158220

[ref37] Banaszynski L. A.; Chen L.; Maynard-Smith L. A.; Ooi A. G. L.; Wandless T. J. A Rapid, Reversible, and Tunable Method to Regulate Protein Function in Living Cells Using Synthetic Small Molecules. Cell 2006, 126 (5), 995–1004. 10.1016/j.cell.2006.07.025. 16959577 PMC3290523

[ref38] Miyamae Y.; Chen L.; Utsugi Y.; Farrants H.; Wandless T. J. A Method for Conditional Regulation of Protein Stability in Native or Near-Native Form. Cell Chem. Biol. 2020, 27 (12), 1573–1581.e3. 10.1016/j.chembiol.2020.09.004. 33007216 PMC7749034

[ref39] Fegan A.; White B.; Carlson J. C. T.; Wagner C. R. Chemically Controlled Protein Assembly: Techniques and Applications. Chem. Rev. 2010, 110 (6), 3315–3336. 10.1021/cr8002888. 20353181

[ref40] Stanton B. Z.; Chory E. J.; Crabtree G. R. Chemically Induced Proximity in Biology and Medicine. Science 2018, 359 (6380), eaao5902 10.1126/science.aao5902. 29590011 PMC6417506

[ref41] Gerry C. J.; Schreiber S. L. Unifying Principles of Bifunctional, Proximity-Inducing Small Molecules. Nat. Chem. Biol. 2020, 16 (4), 369–378. 10.1038/s41589-020-0469-1. 32198490 PMC7312755

[ref42] Ng C. S. C.; Banik S. M. Recent Advances in Induced Proximity Modalities. Curr. Opin. Chem. Biol. 2022, 67, 102107 10.1016/j.cbpa.2021.102107. 35033823

[ref43] Liu J.; Farmer J. D.; Lane W. S.; Friedman J.; Weissman I.; Schreiber S. L. Calcineurin Is a Common Target of Cyclophilin-Cyclosporin A and FKBP-FK506 Complexes. Cell 1991, 66 (4), 807–815. 10.1016/0092-8674(91)90124-H. 1715244

[ref44] Spencer D.; Wandless T.; Schreiber S.; Crabtree G. Controlling Signal Transduction with Synthetic Ligands. Science 1993, 262 (5136), 1019 10.1126/science.7694365. 7694365

[ref45] Liang F.-S.; Ho W. Q.; Crabtree G. R. Engineering the ABA Plant Stress Pathway for Regulation of Induced Proximity. Sci. Signal. 2011, 4 (164), rs2 10.1126/scisignal.2001449. 21406691 PMC3110149

[ref46] Hill Z. B.; Martinko A. J.; Nguyen D. P.; Wells J. A. Human Antibody-Based Chemically Induced Dimerizers for Cell Therapeutic Applications. Nat. Chem. Biol. 2018, 14 (2), 112–117. 10.1038/nchembio.2529. 29200207 PMC6352901

[ref47] Luo Z.; Tzivion G.; Belshaw P. J.; Vavvas D.; Marshall M.; Avruch J. Oligomerization Activates C-Raf-1 through a Ras-Dependent Mechanism. Nature 1996, 383 (6596), 181–185. 10.1038/383181a0. 8774885

[ref48] Spencer D. M.; Belshaw P. J.; Chen L.; Ho S. N.; Randazzo F.; Crabtree G. R.; Schreiber S. L. Functional Analysis of Fas Signaling in Vivo Using Synthetic Inducers of Dimerization. Curr. Biol. 1996, 6 (7), 839–847. 10.1016/S0960-9822(02)00607-3. 8805308

[ref49] Wu C.-Y.; Roybal K. T.; Puchner E. M.; Onuffer J.; Lim W. A. Remote Control of Therapeutic T Cells through a Small Molecule-Gated Chimeric Receptor. Science 2015, 350 (6258), aab4077–aab4077. 10.1126/science.aab4077. 26405231 PMC4721629

[ref50] Maccorkle R. A.; Freeman K. W.; Spencer D. M. Synthetic Activation of Caspases: Artificial Death Switches. Proc. Natl. Acad. Sci. U. S. A. 1998, 95 (7), 3655–3660. 10.1073/pnas.95.7.3655. 9520421 PMC19891

[ref51] Di Stasi A.; Tey S.-K.; Dotti G.; Fujita Y.; Kennedy-Nasser A.; Martinez C.; Straathof K.; Liu E.; Durett A. G.; Grilley B.; Liu H.; Cruz C. R.; Savoldo B.; Gee A. P.; Schindler J.; Krance R. A.; Heslop H. E.; Spencer D. M.; Rooney C. M.; Brenner M. K. Inducible Apoptosis as a Safety Switch for Adoptive Cell Therapy. N. Engl. J. Med. 2011, 365 (18), 1673–1683. 10.1056/NEJMoa1106152. 22047558 PMC3236370

[ref52] Voß S.; Klewer L.; Wu Y.-W. Chemically Induced Dimerization: Reversible and Spatiotemporal Control of Protein Function in Cells. Curr. Opin. Chem. Biol. 2015, 28, 194–201. 10.1016/j.cbpa.2015.09.003. 26431673

[ref53] Rihtar E.; Lebar T.; Lainšček D.; Kores K.; Lešnik S.; Bren U.; Jerala R. Chemically Inducible Split Protein Regulators for Mammalian Cells. Nat. Chem. Biol. 2023, 19 (1), 64–71. 10.1038/s41589-022-01136-x. 36163385

[ref54] Ziegler M. J.; Yserentant K.; Dunsing V.; Middel V.; Gralak A. J.; Pakari K.; Bargstedt J.; Kern C.; Petrich A.; Chiantia S.; Strähle U.; Herten D.-P.; Wombacher R. Mandipropamid as a Chemical Inducer of Proximity for in Vivo Applications. Nat. Chem. Biol. 2022, 18 (1), 64–69. 10.1038/s41589-021-00922-3. 34934192 PMC8709788

[ref55] Crossman S. H.; Janovjak H. Light-Activated Receptor Tyrosine Kinases: Designs and Applications. Curr. Opin. Pharmacol. 2022, 63, 102197 10.1016/j.coph.2022.102197. 35245796

[ref56] Krueger D.; Izquierdo E.; Viswanathan R.; Hartmann J.; Pallares Cartes C.; De Renzis S. Principles and Applications of Optogenetics in Developmental Biology. Development 2019, 146 (20), dev175067 10.1242/dev.175067. 31641044 PMC6914371

[ref57] Zhou X. X.; Chung H. K.; Lam A. J.; Lin M. Z. Optical Control of Protein Activity by Fluorescent Protein Domains. Science 2012, 338 (6108), 810–814. 10.1126/science.1226854. 23139335 PMC3702057

[ref58] Zhou X. X.; Fan L. Z.; Li P.; Shen K.; Lin M. Z. Optical Control of Cell Signaling by Single-Chain Photoswitchable Kinases. Science 2017, 355 (6327), 836–842. 10.1126/science.aah3605. 28232577 PMC5589340

[ref59] Bell M. R.; Engleka M. J.; Malik A.; Strickler J. E. To Fuse or Not to Fuse: What Is Your Purpose?: Protein Fusion Technology. Protein Sci. 2013, 22 (11), 1466–1477. 10.1002/pro.2356. 24038604 PMC3831663

[ref60] Deshaies R. J. Prime Time for PROTACs. Nat. Chem. Biol. 2015, 11 (9), 634–635. 10.1038/nchembio.1887. 26284668

[ref61] Nalawansha D. A.; Crews C. M. PROTACs: An Emerging Therapeutic Modality in Precision Medicine. Cell Chem. Biol. 2020, 27 (8), 998–1014. 10.1016/j.chembiol.2020.07.020. 32795419 PMC9424844

[ref62] Cowan A. D.; Ciulli A. Driving E3 Ligase Substrate Specificity for Targeted Protein Degradation: Lessons from Nature and the Laboratory. Annu. Rev. Biochem. 2022, 91 (1), 295–319. 10.1146/annurev-biochem-032620-104421. 35320687

[ref63] Kiely-Collins H.; Winter G. E.; Bernardes G. J. L. The Role of Reversible and Irreversible Covalent Chemistry in Targeted Protein Degradation. Cell Chem. Biol. 2021, 28 (7), 952–968. 10.1016/j.chembiol.2021.03.005. 33789091

[ref64] Schreiber S. L. The Rise of Molecular Glues. Cell 2021, 184 (1), 3–9. 10.1016/j.cell.2020.12.020. 33417864

[ref65] Mayor-Ruiz C.; Bauer S.; Brand M.; Kozicka Z.; Siklos M.; Imrichova H.; Kaltheuner I. H.; Hahn E.; Seiler K.; Koren A.; Petzold G.; Fellner M.; Bock C.; Müller A. C.; Zuber J.; Geyer M.; Thomä N. H.; Kubicek S.; Winter G. E. Rational Discovery of Molecular Glue Degraders via Scalable Chemical Profiling. Nat. Chem. Biol. 2020, 16 (11), 1199–1207. 10.1038/s41589-020-0594-x. 32747809 PMC7116640

[ref66] Dong G.; Ding Y.; He S.; Sheng C. Molecular Glues for Targeted Protein Degradation: From Serendipity to Rational Discovery. J. Med. Chem. 2021, 64 (15), 10606–10620. 10.1021/acs.jmedchem.1c00895. 34319094

[ref67] Kozicka Z.; Thomä N. H. Haven’t Got a Glue: Protein Surface Variation for the Design of Molecular Glue Degraders. Cell Chem. Biol. 2021, 28 (7), 1032–1047. 10.1016/j.chembiol.2021.04.009. 33930325

[ref68] Alabi S. B.; Crews C. M. Major Advances in Targeted Protein Degradation: PROTACs, LYTACs, and MADTACs. J. Biol. Chem. 2021, 296, 100647 10.1016/j.jbc.2021.100647. 33839157 PMC8131913

[ref69] Takahashi D.; Moriyama J.; Nakamura T.; Miki E.; Takahashi E.; Sato A.; Akaike T.; Itto-Nakama K.; Arimoto H. AUTACs: Cargo-Specific Degraders Using Selective Autophagy. Mol. Cell 2019, 76 (5), 797–810.e10. 10.1016/j.molcel.2019.09.009. 31606272

[ref70] Ji C. H.; Kim H. Y.; Lee M. J.; Heo A. J.; Park D. Y.; Lim S.; Shin S.; Ganipisetti S.; Yang W. S.; Jung C. A.; Kim K. Y.; Jeong E. H.; Park S. H.; Bin Kim S.; Lee S. J.; Na J. E.; Kang J. I.; Chi H. M.; Kim H. T.; Kim Y. K.; Kim B. Y.; Kwon Y. T. The AUTOTAC Chemical Biology Platform for Targeted Protein Degradation via the Autophagy-Lysosome System. Nat. Commun. 2022, 13 (1), 904 10.1038/s41467-022-28520-4. 35173167 PMC8850458

[ref71] Li Z.; Zhu C.; Ding Y.; Fei Y.; Lu B. ATTEC: A Potential New Approach to Target Proteinopathies. Autophagy 2020, 16 (1), 185–187. 10.1080/15548627.2019.1688556. 31690177 PMC6984452

[ref72] Henning N. J.; Boike L.; Spradlin J. N.; Ward C. C.; Liu G.; Zhang E.; Belcher B. P.; Brittain S. M.; Hesse M. J.; Dovala D.; McGregor L. M.; Valdez Misiolek R.; Plasschaert L. W.; Rowlands D. J.; Wang F.; Frank A. O.; Fuller D.; Estes A. R.; Randal K. L.; Panidapu A.; McKenna J. M.; Tallarico J. A.; Schirle M.; Nomura D. K. Deubiquitinase-Targeting Chimeras for Targeted Protein Stabilization. Nat. Chem. Biol. 2022, 18 (4), 412–421. 10.1038/s41589-022-00971-2. 35210618 PMC10125259

[ref73] Siriwardena S. U.; Munkanatta Godage D. N. P.; Shoba V. M.; Lai S.; Shi M.; Wu P.; Chaudhary S. K.; Schreiber S. L.; Choudhary A. Phosphorylation-Inducing Chimeric Small Molecules. J. Am. Chem. Soc. 2020, 142 (33), 14052–14057. 10.1021/jacs.0c05537. 32787262

[ref74] Chen P.-H.; Hu Z.; An E.; Okeke I.; Zheng S.; Luo X.; Gong A.; Jaime-Figueroa S.; Crews C. M. Modulation of Phosphoprotein Activity by Phosphorylation Targeting Chimeras (PhosTACs). ACS Chem. Biol. 2021, 16 (12), 2808–2815. 10.1021/acschembio.1c00693. 34780684 PMC10437008

[ref75] Wang W. W.; Chen L.-Y.; Wozniak J. M.; Jadhav A. M.; Anderson H.; Malone T. E.; Parker C. G. Targeted Protein Acetylation in Cells Using Heterobifunctional Molecules. J. Am. Chem. Soc. 2021, 143 (40), 16700–16708. 10.1021/jacs.1c07850. 34592107 PMC10793965

[ref76] Caianiello D. F.; Zhang M.; Ray J. D.; Howell R. A.; Swartzel J. C.; Branham E. M. J.; Chirkin E.; Sabbasani V. R.; Gong A. Z.; McDonald D. M.; Muthusamy V.; Spiegel D. A. Bifunctional Small Molecules That Mediate the Degradation of Extracellular Proteins. Nat. Chem. Biol. 2021, 17 (9), 947–953. 10.1038/s41589-021-00851-1. 34413525

[ref77] Banik S. M.; Pedram K.; Wisnovsky S.; Ahn G.; Riley N. M.; Bertozzi C. R. Lysosome-Targeting Chimaeras for Degradation of Extracellular Proteins. Nature 2020, 584 (7820), 291–297. 10.1038/s41586-020-2545-9. 32728216 PMC7727926

[ref78] Cotton A. D.; Nguyen D. P.; Gramespacher J. A.; Seiple I. B.; Wells J. A. Development of Antibody-Based PROTACs for the Degradation of the Cell-Surface Immune Checkpoint Protein PD-L1. J. Am. Chem. Soc. 2021, 143 (2), 593–598. 10.1021/jacs.0c10008. 33395526 PMC8154509

[ref79] Kargbo R. B. Emerging Proteolysis Targeting Antibodies (PROTABs) for Application in Cancer Therapy. ACS Med. Chem. Lett. 2022, 13 (12), 1833–1834. 10.1021/acsmedchemlett.2c00458. 36518693 PMC9743953

[ref80] Zhang H.; Han Y.; Yang Y.; Lin F.; Li K.; Kong L.; Liu H.; Dang Y.; Lin J.; Chen P. R. Covalently Engineered Nanobody Chimeras for Targeted Membrane Protein Degradation. J. Am. Chem. Soc. 2021, 143 (40), 16377–16382. 10.1021/jacs.1c08521. 34596400

[ref81] Pance K.; Gramespacher J. A.; Byrnes J. R.; Salangsang F.; Serrano J.-A. C.; Cotton A. D.; Steri V.; Wells J. A. Modular Cytokine Receptor-Targeting Chimeras for Targeted Degradation of Cell Surface and Extracellular Proteins. Nat. Biotechnol. 2022, 10.1038/s41587-022-01456-2. PMC9931583 36138170

[ref82] Watkins A. M.; Arora P. S. Structure-Based Inhibition of Protein–Protein Interactions. Eur. J. Med. Chem. 2015, 94, 480–488. 10.1016/j.ejmech.2014.09.047. 25253637 PMC4362920

[ref83] Lu H.; Zhou Q.; He J.; Jiang Z.; Peng C.; Tong R.; Shi J. Recent Advances in the Development of Protein–Protein Interactions Modulators: Mechanisms and Clinical Trials. Signal Transduct. Target. Ther. 2020, 5 (1), 213 10.1038/s41392-020-00315-3. 32968059 PMC7511340

[ref84] Stevens A. J.; Brown Z. Z.; Shah N. H.; Sekar G.; Cowburn D.; Muir T. W. Design of a Split Intein with Exceptional Protein Splicing Activity. J. Am. Chem. Soc. 2016, 138 (7), 2162–2165. 10.1021/jacs.5b13528. 26854538 PMC4894280

[ref85] Burton A. J.; Haugbro M.; Parisi E.; Muir T. W. Live-Cell Protein Engineering with an Ultra-Short Split Intein. Proc. Natl. Acad. Sci. U. S. A. 2020, 117 (22), 12041–12049. 10.1073/pnas.2003613117. 32424098 PMC7275667

[ref86] Zabin I.; Villarejo M. R. Protein Complementation. Annu. Rev. Biochem. 1975, 44 (1), 295–313. 10.1146/annurev.bi.44.070175.001455. 124547

[ref87] Michnick S. W.; Ear P. H.; Manderson E. N.; Remy I.; Stefan E. Universal Strategies in Research and Drug Discovery Based on Protein-Fragment Complementation Assays. Nat. Rev. Drug Discovery 2007, 6 (7), 569–582. 10.1038/nrd2311. 17599086

[ref88] Quijano-Rubio A.; Bhuiyan A. M.; Yang H.; Leung I.; Bello E.; Ali L. R.; Zhangxu K.; Perkins J.; Chun J.-H.; Wang W.; Lajoie M. J.; Ravichandran R.; Kuo Y.-H.; Dougan S. K.; Riddell S. R.; Spangler J. B.; Dougan M.; Silva D.-A.; Baker D. A Split, Conditionally Active Mimetic of IL-2 Reduces the Toxicity of Systemic Cytokine Therapy. Nat. Biotechnol. 2022, 10.1038/s41587-022-01510-z. PMC10110466 36316485

[ref89] Silva D.-A.; Yu S.; Ulge U. Y.; Spangler J. B.; Jude K. M.; Labão-Almeida C.; Ali L. R.; Quijano-Rubio A.; Ruterbusch M.; Leung I.; Biary T.; Crowley S. J.; Marcos E.; Walkey C. D.; Weitzner B. D.; Pardo-Avila F.; Castellanos J.; Carter L.; Stewart L.; Riddell S. R.; Pepper M.; Bernardes G. J. L.; Dougan M.; Garcia K. C.; Baker D. De Novo Design of Potent and Selective Mimics of IL-2 and IL-15. Nature 2019, 565 (7738), 186–191. 10.1038/s41586-018-0830-7. 30626941 PMC6521699

[ref90] Mok B. Y.; de Moraes M. H.; Zeng J.; Bosch D. E.; Kotrys A. V.; Raguram A.; Hsu F.; Radey M. C.; Peterson S. B.; Mootha V. K.; Mougous J. D.; Liu D. R. A Bacterial Cytidine Deaminase Toxin Enables CRISPR-Free Mitochondrial Base Editing. Nature 2020, 583 (7817), 631–637. 10.1038/s41586-020-2477-4. 32641830 PMC7381381

[ref91] Ge Y.; Ramirez D. H.; Yang B.; D’Souza A. K.; Aonbangkhen C.; Wong S.; Woo C. M. Target Protein Deglycosylation in Living Cells by a Nanobody-Fused Split O-GlcNAcase. Nat. Chem. Biol. 2021, 17 (5), 593–600. 10.1038/s41589-021-00757-y. 33686291 PMC8085020

[ref92] Cho K. F.; Branon T. C.; Rajeev S.; Svinkina T.; Udeshi N. D.; Thoudam T.; Kwak C.; Rhee H.-W.; Lee I.-K.; Carr S. A.; Ting A. Y. Split-TurboID Enables Contact-Dependent Proximity Labeling in Cells. Proc. Natl. Acad. Sci. U. S. A. 2020, 117 (22), 12143–12154. 10.1073/pnas.1919528117. 32424107 PMC7275672

[ref93] Bennett M. J.; Choe S.; Eisenberg D. Domain Swapping: Entangling Alliances between Proteins. Proc. Natl. Acad. Sci. U. S. A. 1994, 91 (8), 3127–3131. 10.1073/pnas.91.8.3127. 8159715 PMC43528

[ref94] Stroud J. C.; Liu C.; Teng P. K.; Eisenberg D. Toxic Fibrillar Oligomers of Amyloid-β Have Cross-β Structure. Proc. Natl. Acad. Sci. U. S. A. 2012, 109 (20), 7717–7722. 10.1073/pnas.1203193109. 22547798 PMC3356606

[ref95] Ha J.-H.; Karchin J. M.; Walker-Kopp N.; Castañeda C. A.; Loh S. N. Engineered Domain Swapping as an On/Off Switch for Protein Function. Chem. Biol. 2015, 22 (10), 1384–1393. 10.1016/j.chembiol.2015.09.007. 26496687 PMC4621486

[ref96] Reis J. M.; Burns D. C.; Woolley G. A. Optical Control of Protein–Protein Interactions via Blue Light-Induced Domain Swapping. Biochemistry 2014, 53 (30), 5008–5016. 10.1021/bi500622x. 25003701 PMC4372075

[ref97] Ha J.-H.; Loh S. N. Protein Conformational Switches: From Nature to Design. Chem.—Eur. J. 2012, 18 (26), 7984–7999. 10.1002/chem.201200348. 22688954 PMC3404493

[ref98] Hakala T. A.; Yates E. V.; Challa P. K.; Toprakcioglu Z.; Nadendla K.; Matak-Vinkovic D.; Dobson C. M.; Martínez R.; Corzana F.; Knowles T. P. J.; Bernardes G. J. L. Accelerating Reaction Rates of Biomolecules by Using Shear Stress in Artificial Capillary Systems. J. Am. Chem. Soc. 2021, 143 (40), 16401–16410. 10.1021/jacs.1c03681. 34606279 PMC8517977

[ref99] Murtaugh M. L.; Fanning S. W.; Sharma T. M.; Terry A. M.; Horn J. R. A Combinatorial Histidine Scanning Library Approach to Engineer Highly PH-Dependent Protein Switches: Engineering PH-Sensitive Protein Switches. Protein Sci. 2011, 20 (9), 1619–1631. 10.1002/pro.696. 21766385 PMC3190156

[ref100] Strauch E.-M.; Fleishman S. J.; Baker D. Computational Design of a PH-Sensitive IgG Binding Protein. Proc. Natl. Acad. Sci. U. S. A. 2014, 111 (2), 675–680. 10.1073/pnas.1313605111. 24381156 PMC3896196

[ref101] Boyken S. E.; Benhaim M. A.; Busch F.; Jia M.; Bick M. J.; Choi H.; Klima J. C.; Chen Z.; Walkey C.; Mileant A.; Sahasrabuddhe A.; Wei K. Y.; Hodge E. A.; Byron S.; Quijano-Rubio A.; Sankaran B.; King N. P.; Lippincott-Schwartz J.; Wysocki V. H.; Lee K. K.; Baker D. De Novo Design of Tunable, PH-Driven Conformational Changes. Science 2019, 364 (6441), 658–664. 10.1126/science.aav7897. 31097662 PMC7072037

[ref102] Gera N.; Hill A. B.; White D. P.; Carbonell R. G.; Rao B. M. Design of PH Sensitive Binding Proteins from the Hyperthermophilic Sso7d Scaffold. PLoS One 2012, 7 (11), e48928 10.1371/journal.pone.0048928. 23145025 PMC3492137

[ref103] Jiang B.; Liu X.; Yang C.; Yang Z.; Luo J.; Kou S.; Liu K.; Sun F. Injectable, Photoresponsive Hydrogels for Delivering Neuroprotective Proteins Enabled by Metal-Directed Protein Assembly. Sci. Adv. 2020, 6 (41), eabc4824 10.1126/sciadv.abc4824. 33036976 PMC7546710

[ref104] Huerta-López C.; Alegre-Cebollada J. Protein Hydrogels: The Swiss Army Knife for Enhanced Mechanical and Bioactive Properties of Biomaterials. Nanomaterials 2021, 11 (7), 1656 10.3390/nano11071656. 34202469 PMC8307158

[ref105] Bozovic O.; Jankovic B.; Hamm P. Using Azobenzene Photocontrol to Set Proteins in Motion. Nat. Rev. Chem. 2022, 6 (2), 112–124. 10.1038/s41570-021-00338-6. 37117294

[ref106] Mart R. J.; Allemann R. K. Azobenzene Photocontrol of Peptides and Proteins. Chem. Commun. 2016, 52 (83), 12262–12277. 10.1039/C6CC04004G. 27541361

[ref107] Wang Z. A.; Cole P. A. The Chemical Biology of Reversible Lysine Post-Translational Modifications. Cell Chem. Biol. 2020, 27 (8), 953–969. 10.1016/j.chembiol.2020.07.002. 32698016 PMC7487139

[ref108] Schnell J. D.; Hicke L. Non-Traditional Functions of Ubiquitin and Ubiquitin-Binding Proteins. J. Biol. Chem. 2003, 278 (38), 35857–35860. 10.1074/jbc.R300018200. 12860974

[ref109] Andrés M.; García-Gomis D.; Ponte I.; Suau P.; Roque A. Histone H1 Post-Translational Modifications: Update and Future Perspectives. Int. J. Mol. Sci. 2020, 21 (16), 5941 10.3390/ijms21165941. 32824860 PMC7460583

[ref110] Hall J. E. Guyton and Hall Textbook of Medical Physiology, 13th ed.; Elsevier: Philadelphia, PA, 2016.

[ref111] Flexner C. HIV-Protease Inhibitors. N. Engl. J. Med. 1998, 338 (18), 1281–1293. 10.1056/NEJM199804303381808. 9562584

[ref112] Walsh P. N.; Ahmad S. S. Proteases in Blood Clotting. Essays Biochem 2002, 38, 95–111. 10.1042/bse0380095. 12463164

[ref113] Johnson R. J.; Lin S. R.; Raines R. T. A Ribonuclease Zymogen Activated by the NS3 Protease of the Hepatitis C Virus. FEBS J. 2006, 273 (23), 5457–5465. 10.1111/j.1742-4658.2006.05536.x. 17116245

[ref114] Yeruva T.; Lee C. H. Enzyme Responsive Delivery of Anti-Retroviral Peptide via Smart Hydrogel. AAPS PharmSciTech 2022, 23 (7), 234 10.1208/s12249-022-02391-w. 36002705

[ref115] Yachnin B. J.; Azouz L. R.; White R. E.; Minetti C. A. S. A.; Remeta D. P.; Tan V. M.; Drake J. M.; Khare S. D. Massively Parallel, Computationally Guided Design of a Proenzyme. Proc. Natl. Acad. Sci. U. S. A. 2022, 119 (15), e2116097119 10.1073/pnas.2116097119. 35377786 PMC9169645

[ref116] Yeruva T. Smart Hydrogel for Enzyme Responsive Vaginal Delivery of Anti-HIV Peptide Therapeutics; University of Missouri-Kansas City, 2021.

[ref117] Puskas J.; Skrombolas D.; Sedlacek A.; Lord E.; Sullivan M.; Frelinger J. Development of an Attenuated Interleukin-2 Fusion Protein That Can Be Activated by Tumour-Expressed Proteases: Protease-Activated Cytokines. Immunology 2011, 133 (2), 206–220. 10.1111/j.1365-2567.2011.03428.x. 21426339 PMC3088983

[ref118] Trang V. H.; Zhang X.; Yumul R. C.; Zeng W.; Stone I. J.; Wo S. W.; Dominguez M. M.; Cochran J. H.; Simmons J. K.; Ryan M. C.; Lyon R. P.; Senter P. D.; Levengood M. R. A Coiled-Coil Masking Domain for Selective Activation of Therapeutic Antibodies. Nat. Biotechnol. 2019, 37 (7), 761–765. 10.1038/s41587-019-0135-x. 31133742

[ref119] Assi H. H.; Wong C.; Tipton K. A.; Mei L.; Wong K.; Razo J.; Chan C.; Howng B.; Sagert J.; Krimm M.; Diep L.; Jang A.; Nguyen M. T.; Lapuyade N.; Singson V.; Villanueva R.; Paidhungat M.; Liu S.; Rangan V.; Vasiljeva O.; West J. W.; Richardson J. H.; Irving B.; Daniel D.; Belvin M.; Kavanaugh W. M. Conditional PD-1/PD-L1 Probody Therapeutics Induce Comparable Antitumor Immunity but Reduced Systemic Toxicity Compared with Traditional Anti–PD-1/PD-L1 Agents. Cancer Immunol. Res. 2021, 9 (12), 1451–1464. 10.1158/2326-6066.CIR-21-0031. 34635485 PMC9414278

[ref120] Desnoyers L. R.; Vasiljeva O.; Richardson J. H.; Yang A.; Menendez E. E. M.; Liang T. W.; Wong C.; Bessette P. H.; Kamath K.; Moore S. J.; Sagert J. G.; Hostetter D. R.; Han F.; Gee J.; Flandez J.; Markham K.; Nguyen M.; Krimm M.; Wong K. R.; Liu S.; Daugherty P. S.; West J. W.; Lowman H. B. Tumor-Specific Activation of an EGFR-Targeting Probody Enhances Therapeutic Index. Sci. Transl. Med. 2013, 5 (207), 207ra144 10.1126/scitranslmed.3006682. 24132639

[ref121] Glibowicka M.; He S.; Deber C. M. Enhanced Proteolytic Resistance of Cationic Antimicrobial Peptides through Lysine Side Chain Analogs and Cyclization. Biochem. Biophys. Res. Commun. 2022, 612, 105–109. 10.1016/j.bbrc.2022.04.113. 35512459

[ref122] Charych D. H.; Hoch U.; Langowski J. L.; Lee S. R.; Addepalli M. K.; Kirk P. B.; Sheng D.; Liu X.; Sims P. W.; VanderVeen L. A.; Ali C. F.; Chang T. K.; Konakova M.; Pena R. L.; Kanhere R. S.; Kirksey Y. M.; Ji C.; Wang Y.; Huang J.; Sweeney T. D.; Kantak S. S.; Doberstein S. K. NKTR-214, an Engineered Cytokine with Biased IL2 Receptor Binding, Increased Tumor Exposure, and Marked Efficacy in Mouse Tumor Models. Clin. Cancer Res. 2016, 22 (3), 680–690. 10.1158/1078-0432.CCR-15-1631. 26832745

[ref123] Boike L.; Henning N. J.; Nomura D. K. Advances in Covalent Drug Discovery. Nat. Rev. Drug Discovery 2022, 21, 881–898. 10.1038/s41573-022-00542-z. 36008483 PMC9403961

[ref124] Nussinov R.; Tsai C.-J. The Design of Covalent Allosteric Drugs. Annu. Rev. Pharmacol. Toxicol. 2015, 55 (1), 249–267. 10.1146/annurev-pharmtox-010814-124401. 25149918

[ref125] Lu S.; Zhang J. Designed Covalent Allosteric Modulators: An Emerging Paradigm in Drug Discovery. Drug Discovery Today 2017, 22 (2), 447–453. 10.1016/j.drudis.2016.11.013. 27888140

[ref126] Zheng Q.; Woehl J. L.; Kitamura S.; Santos-Martins D.; Smedley C. J.; Li G.; Forli S.; Moses J. E.; Wolan D. W.; Sharpless K. B. SuFEx-Enabled, Agnostic Discovery of Covalent Inhibitors of Human Neutrophil Elastase. Proc. Natl. Acad. Sci. U. S. A. 2019, 116 (38), 18808–18814. 10.1073/pnas.1909972116. 31484779 PMC6754619

[ref127] Walsh C. T. Suicide Substrates: Mechanism-Based Enzyme Inactivators with Therapeutic Potential. Trends Biochem. Sci. 1983, 8 (7), 254–257. 10.1016/0968-0004(83)90352-3.

[ref128] Singh J.; Petter R. C.; Baillie T. A.; Whitty A. The Resurgence of Covalent Drugs. Nat. Rev. Drug Discovery 2011, 10 (4), 307–317. 10.1038/nrd3410. 21455239

[ref129] Taylor R. J.; Geeson M. B.; Journeaux T.; Bernardes G. J. L. Chemical and Enzymatic Methods for Post-Translational Protein–Protein Conjugation. J. Am. Chem. Soc. 2022, 144 (32), 14404–14419. 10.1021/jacs.2c00129. 35912579 PMC9389620

[ref130] Taylor R. J.; Aguilar Rangel M.; Geeson M. B.; Sormanni P.; Vendruscolo M.; Bernardes G. J. L. π-Clamp-Mediated Homo- and Heterodimerization of Single-Domain Antibodies via Site-Specific Homobifunctional Conjugation. J. Am. Chem. Soc. 2022, 144 (29), 13026–13031. 10.1021/jacs.2c04747. 35834748 PMC9335888

[ref131] Rehm F. B. H.; Tyler T. J.; Veer S. J.; Craik D. J.; Durek T. Enzymatic C-to-C Protein Ligation. Angew. Chem., Int. Ed. 2022, 61 (11), e202116672 10.1002/anie.202116672. PMC9303898 35018698

[ref132] Szijj P.; Chudasama V. The Renaissance of Chemically Generated Bispecific Antibodies. Nat. Rev. Chem. 2021, 5 (2), 78–92. 10.1038/s41570-020-00241-6. 37117612

[ref133] Poo W.-J.; Hartshorne D. J. Actin Crosslinked with Glutaraldehyde: Evidence to Suggest an Active Role for Actin in the Regulatory Mechanism. Biochem. Biophys. Res. Commun. 1976, 70 (2), 406–412. 10.1016/0006-291X(76)91060-3. 132931

[ref134] Adamiak K.; Sionkowska A. Current Methods of Collagen Cross-Linking: Review. Int. J. Biol. Macromol. 2020, 161, 550–560. 10.1016/j.ijbiomac.2020.06.075. 32534089

[ref135] Schiffner T.; Pallesen J.; Russell R. A.; Dodd J.; de Val N.; LaBranche C. C.; Montefiori D.; Tomaras G. D.; Shen X.; Harris S. L.; Moghaddam A. E.; Kalyuzhniy O.; Sanders R. W.; McCoy L. E.; Moore J. P.; Ward A. B.; Sattentau Q. J. Structural and Immunologic Correlates of Chemically Stabilized HIV-1 Envelope Glycoproteins. PLOS Pathog 2018, 14 (5), e1006986 10.1371/journal.ppat.1006986. 29746590 PMC5944921

[ref136] Sarmiento C.; Camarero J. A. Biotechnological Applications of Protein Splicing. Curr. Protein Pept. Sci. 2019, 20 (5), 408–424. 10.2174/1389203720666190208110416. 30734675 PMC7135711

[ref137] Woods D.; Vangaveti S.; Egbanum I.; Sweeney A. M.; Li Z.; Bacot-Davis V.; LeSassier D. S.; Stanger M.; Hardison G. E.; Li H.; Belfort M.; Lennon C. W. Conditional DnaB Protein Splicing Is Reversibly Inhibited by Zinc in Mycobacteria. mBio 2020, 11 (4), e01403-20 10.1128/mBio.01403-20. 32665276 PMC7360933

[ref138] Gramespacher J. A.; Stevens A. J.; Nguyen D. P.; Chin J. W.; Muir T. W. Intein Zymogens: Conditional Assembly and Splicing of Split Inteins via Targeted Proteolysis. J. Am. Chem. Soc. 2017, 139 (24), 8074–8077. 10.1021/jacs.7b02618. 28562027 PMC5533455

[ref139] Topilina N. I.; Mills K. V. Recent Advances in in Vivo Applications of Intein-Mediated Protein Splicing. Mob. DNA 2014, 5 (1), 5 10.1186/1759-8753-5-5. 24490831 PMC3922620

[ref140] Di Ventura B.; Mootz H. D. Switchable Inteins for Conditional Protein Splicing. Biol. Chem. 2019, 400 (4), 467–475. 10.1515/hsz-2018-0309. 30226200

[ref141] Luo J.; Liu Q.; Morihiro K.; Deiters A. Small-Molecule Control of Protein Function through Staudinger Reduction. Nat. Chem. 2016, 8 (11), 1027–1034. 10.1038/nchem.2573. 27768095 PMC5119652

[ref142] Chintala S.; Friedman S. H. A Light Activated Glucagon Trimer with Resistance to Fibrillation. ACS Biomater. Sci. Eng. 2021, 7 (4), 1506–1514. 10.1021/acsbiomaterials.1c00031. 33703874 PMC8906801

[ref143] Nadendla K.; Friedman S. H. Light Control of Protein Solubility Through Isoelectric Point Modulation. J. Am. Chem. Soc. 2017, 139 (49), 17861–17869. 10.1021/jacs.7b08465. 29192764 PMC6362458

[ref144] Nadendla K.; Sarode B. R.; Friedman S. H. Hydrophobic Tags for Highly Efficient Light-Activated Protein Release. Mol. Pharmaceutics 2019, 16 (7), 2922–2928. 10.1021/acs.molpharmaceut.9b00140. PMC7050909 31117739

[ref145] Zubi Y. S.; Seki K.; Li Y.; Hunt A. C.; Liu B.; Roux B.; Jewett M. C.; Lewis J. C. Metal-Responsive Regulation of Enzyme Catalysis Using Genetically Encoded Chemical Switches. Nat. Commun. 2022, 13 (1), 1864 10.1038/s41467-022-29239-y. 35387988 PMC8987029

[ref146] Chang J.; Cai W.; Liang C.; Tang Q.; Chen X.; Jiang Y.; Mao L.; Wang M. Enzyme-Instructed Activation of Pro-Protein Therapeutics In Vivo. J. Am. Chem. Soc. 2019, 141 (45), 18136–18141. 10.1021/jacs.9b08669. 31589435

[ref147] Sabatino V.; Unnikrishnan V. B.; Bernardes G. J. L. Transition Metal Mediated Bioorthogonal Release. Chem. Catal 2022, 2 (1), 39–51. 10.1016/j.checat.2021.12.007.

[ref148] Oliveira B. L.; Stenton B. J.; Unnikrishnan V. B.; de Almeida C. R.; Conde J.; Negrão M.; Schneider F. S. S.; Cordeiro C.; Ferreira M. G.; Caramori G. F.; Domingos J. B.; Fior R.; Bernardes G. J. L. Platinum-Triggered Bond-Cleavage of Pentynoyl Amide and *N* -Propargyl Handles for Drug-Activation. J. Am. Chem. Soc. 2020, 142 (24), 10869–10880. 10.1021/jacs.0c01622. 32456416 PMC7304066

[ref149] Ge Y.; Fan X.; Chen P. R. A Genetically Encoded Multifunctional Unnatural Amino Acid for Versatile Protein Manipulations in Living Cells. Chem. Sci. 2016, 7 (12), 7055–7060. 10.1039/C6SC02615J. 28451140 PMC5355830

[ref150] Li J.; Jia S.; Chen P. R. Diels-Alder Reaction-Triggered Bioorthogonal Protein Decaging in Living Cells. Nat. Chem. Biol. 2014, 10 (12), 1003–1005. 10.1038/nchembio.1656. 25362360

[ref151] Arbely E.; Torres-Kolbus J.; Deiters A.; Chin J. W. Photocontrol of Tyrosine Phosphorylation in Mammalian Cells via Genetic Encoding of Photocaged Tyrosine. J. Am. Chem. Soc. 2012, 134 (29), 11912–11915. 10.1021/ja3046958. 22758385

[ref152] Zhang G.; Li J.; Xie R.; Fan X.; Liu Y.; Zheng S.; Ge Y.; Chen P. R. Bioorthogonal Chemical Activation of Kinases in Living Systems. ACS Cent. Sci. 2016, 2 (5), 325–331. 10.1021/acscentsci.6b00024. 27280167 PMC4882735

[ref153] Li J.; Yu J.; Zhao J.; Wang J.; Zheng S.; Lin S.; Chen L.; Yang M.; Jia S.; Zhang X.; Chen P. R. Palladium-Triggered Deprotection Chemistry for Protein Activation in Living Cells. Nat. Chem. 2014, 6 (4), 352–361. 10.1038/nchem.1887. 24651204

[ref154] Peters F. B.; Brock A.; Wang J.; Schultz P. G. Photocleavage of the Polypeptide Backbone by 2-Nitrophenylalanine. Chem. Biol. 2009, 16 (2), 148–152. 10.1016/j.chembiol.2009.01.013. 19246005 PMC2714363

[ref155] Wang J.; Liu Y.; Liu Y.; Zheng S.; Wang X.; Zhao J.; Yang F.; Zhang G.; Wang C.; Chen P. R. Time-Resolved Protein Activation by Proximal Decaging in Living Systems. Nature 2019, 569 (7757), 509–513. 10.1038/s41586-019-1188-1. 31068699

[ref156] Lin S.; He D.; Long T.; Zhang S.; Meng R.; Chen P. R. Genetically Encoded Cleavable Protein Photo-Cross-Linker. J. Am. Chem. Soc. 2014, 136 (34), 11860–11863. 10.1021/ja504371w. 25084056

[ref157] Yang Y.; Song H.; He D.; Zhang S.; Dai S.; Xie X.; Lin S.; Hao Z.; Zheng H.; Chen P. R. Genetically Encoded Releasable Photo-Cross-Linking Strategies for Studying Protein–Protein Interactions in Living Cells. Nat. Protoc. 2017, 12 (10), 2147–2168. 10.1038/nprot.2017.090. 28933779

[ref158] Tian Y.; Jacinto M. P.; Zeng Y.; Yu Z.; Qu J.; Liu W. R.; Lin Q. Genetically Encoded 2-Aryl-5-Carboxytetrazoles for Site-Selective Protein Photo-Cross-Linking. J. Am. Chem. Soc. 2017, 139 (17), 6078–6081. 10.1021/jacs.7b02615. 28422494 PMC5423124

[ref159] Podewin T.; Ast J.; Broichhagen J.; Fine N. H. F.; Nasteska D.; Leippe P.; Gailer M.; Buenaventura T.; Kanda N.; Jones B. J.; M’Kadmi C.; Baneres J.-L.; Marie J.; Tomas A.; Trauner D.; Hoffmann-Röder A.; Hodson D. J. Conditional and Reversible Activation of Class A and B G Protein-Coupled Receptors Using Tethered Pharmacology. ACS Cent. Sci. 2018, 4 (2), 166–179. 10.1021/acscentsci.7b00237. 29532016 PMC5832994

[ref160] Leippe P.; Koehler Leman J.; Trauner D. Specificity and Speed: Tethered Photopharmacology. Biochemistry 2017, 56 (39), 5214–5220. 10.1021/acs.biochem.7b00687. 28876905

[ref161] Schöneberg T.; Kleinau G.; Brüser A. What Are They Waiting for?—Tethered Agonism in G Protein-Coupled Receptors. Pharmacol. Res. 2016, 108, 9–15. 10.1016/j.phrs.2016.03.027. 27095083

[ref162] Gianneschi N. C.; Ghadiri M. R. Design of Molecular Logic Devices Based on a Programmable DNA-Regulated Semisynthetic Enzyme. Angew. Chem., Int. Ed. 2007, 46 (21), 3955–3958. 10.1002/anie.200700047. PMC2790070 17427900

[ref163] Mukherjee P.; Leman L. J.; Griffin J. H.; Ghadiri M. R. Design of a DNA-Programmed Plasminogen Activator. J. Am. Chem. Soc. 2018, 140 (45), 15516–15524. 10.1021/jacs.8b10166. 30347143 PMC7115153

[ref164] Saghatelian A.; Guckian K. M.; Thayer D. A.; Ghadiri M. R. DNA Detection and Signal Amplification via an Engineered Allosteric Enzyme. J. Am. Chem. Soc. 2003, 125 (2), 344–345. 10.1021/ja027885u. 12517141 PMC2453066

[ref165] Zhao Z.; Ukidve A.; Kim J.; Mitragotri S. Targeting Strategies for Tissue-Specific Drug Delivery. Cell 2020, 181 (1), 151–167. 10.1016/j.cell.2020.02.001. 32243788

[ref166] Cronican J. J.; Thompson D. B.; Beier K. T.; McNaughton B. R.; Cepko C. L.; Liu D. R. Potent Delivery of Functional Proteins into Mammalian Cells *in Vitro* and *in Vivo* Using a Supercharged Protein. ACS Chem. Biol. 2010, 5 (8), 747–752. 10.1021/cb1001153. 20545362 PMC2924640

[ref167] Schneider A. F. L.; Kithil M.; Cardoso M. C.; Lehmann M.; Hackenberger C. P. R. Cellular Uptake of Large Biomolecules Enabled by Cell-Surface-Reactive Cell-Penetrating Peptide Additives. Nat. Chem. 2021, 13 (6), 530–539. 10.1038/s41557-021-00661-x. 33859390

[ref168] Patel S. G.; Sayers E. J.; He L.; Narayan R.; Williams T. L.; Mills E. M.; Allemann R. K.; Luk L. Y. P.; Jones A. T.; Tsai Y.-H. Cell-Penetrating Peptide Sequence and Modification Dependent Uptake and Subcellular Distribution of Green Florescent Protein in Different Cell Lines. Sci. Rep. 2019, 9 (1), 6298 10.1038/s41598-019-42456-8. 31000738 PMC6472342

[ref169] Ramirez D. H.; Aonbangkhen C.; Wu H.-Y.; Naftaly J. A.; Tang S.; O’Meara T. R.; Woo C. M. Engineering a Proximity-Directed O-GlcNAc Transferase for Selective Protein O-GlcNAcylation in Cells. ACS Chem. Biol. 2020, 15 (4), 1059–1066. 10.1021/acschembio.0c00074. 32119511 PMC7296736

[ref170] Song W.; Yu Z.; Madden M. M.; Lin Q. A Bioorthogonal Chemistry Strategy for Probing Protein Lipidation in Live Cells. Mol. Biosyst. 2010, 6 (9), 1576–1578. 10.1039/c003470c. 20436975 PMC2922461

[ref171] Rudd A. K.; Brea R. J.; Devaraj N. K. Amphiphile-Mediated Depalmitoylation of Proteins in Living Cells. J. Am. Chem. Soc. 2018, 140 (50), 17374–17378. 10.1021/jacs.8b10806. 30516377

[ref172] Wesalo J. S.; Luo J.; Morihiro K.; Liu J.; Deiters A. Phosphine-Activated Lysine Analogues for Fast Chemical Control of Protein Subcellular Localization and Protein SUMOylation. ChemBioChem. 2020, 21 (1–2), 141–148. 10.1002/cbic.201900464. 31664790 PMC6980333

[ref173] Mondal P.; Khamo J. S.; Krishnamurthy V. V.; Cai Q.; Zhang K. Drive the Car(Go)s—New Modalities to Control Cargo Trafficking in Live Cells. Front. Mol. Neurosci 2017, 10.3389/fnmol.2017.00004. PMC5247435 28163671

[ref174] Steketee M. B.; Moysidis S. N.; Jin X.-L.; Weinstein J. E.; Pita-Thomas W.; Raju H. B.; Iqbal S.; Goldberg J. L. Nanoparticle-Mediated Signaling Endosome Localization Regulates Growth Cone Motility and Neurite Growth. Proc. Natl. Acad. Sci. U. S. A. 2011, 108 (47), 19042–19047. 10.1073/pnas.1019624108. 22065745 PMC3223462

[ref175] Fan X.; Geisberg J. V.; Wong K. H.; Jin Y. Conditional Depletion of Nuclear Proteins by the Anchor Away System. In Current Protocols in Molecular Biology; Ausubel F. M., Brent R., Kingston R. E., Moore D. D., Seidman J. G., Smith J. A., Struhl K., Eds.; John Wiley & Sons, Inc.: Hoboken, NJ, USA, 2011. 10.1002/0471142727.mb1310bs93. PMC3076635 21225637

[ref176] Geda P.; Patury S.; Ma J.; Bharucha N.; Dobry C. J.; Lawson S. K.; Gestwicki J. E.; Kumar A. A Small Molecule-Directed Approach to Control Protein Localization and Function. Yeast 2008, 25 (8), 577–594. 10.1002/yea.1610. 18668531

[ref177] Levskaya A.; Weiner O. D.; Lim W. A.; Voigt C. A. Spatiotemporal Control of Cell Signalling Using a Light-Switchable Protein Interaction. Nature 2009, 461 (7266), 997–1001. 10.1038/nature08446. 19749742 PMC2989900

[ref178] Black K. A.; Priftis D.; Perry S. L.; Yip J.; Byun W. Y.; Tirrell M. Protein Encapsulation via Polypeptide Complex Coacervation. ACS Macro Lett. 2014, 3 (10), 1088–1091. 10.1021/mz500529v. 35610798

[ref179] Tomaszewski J. E.; Schwarzenbach R. P.; Sander M. Protein Encapsulation by Humic Substances. Environ. Sci. Technol. 2011, 45 (14), 6003–6010. 10.1021/es200663h. 21678916

[ref180] Colletier J.-P.; Chaize B.; Winterhalter M.; Fournier D. Protein Encapsulation in Liposomes: Efficiency Depends on Interactions between Protein and Phospholipid Bilayer. BMC Biotechnol 2002, 2 (1), 9 10.1186/1472-6750-2-9. 12003642 PMC113741

[ref181] Hanato J.; Kuriyama K.; Mizumoto T.; Debari K.; Hatanaka J.; Onoue S.; Yamada S. Liposomal Formulations of Glucagon-like Peptide-1: Improved Bioavailability and Anti-Diabetic Effect. Int. J. Pharm. 2009, 382 (1–2), 111–116. 10.1016/j.ijpharm.2009.08.013. 19698772

[ref182] Spangler R. S. Insulin Administration via Liposomes. Diabetes Care 1990, 13 (9), 911–922. 10.2337/diacare.13.9.911. 2226109

[ref183] Wong C. Y.; Al-Salami H.; Dass C. R. Recent Advancements in Oral Administration of Insulin-Loaded Liposomal Drug Delivery Systems for Diabetes Mellitus. Int. J. Pharm. 2018, 549 (1–2), 201–217. 10.1016/j.ijpharm.2018.07.041. 30071309

[ref184] de Souza Von Zuben E.; Eloy J. O.; Araujo V. H. S.; Gremião M. P. D.; Chorilli M. Insulin-Loaded Liposomes Functionalized with Cell-Penetrating Peptides: Influence on Drug Release and Permeation through Porcine Nasal Mucosa. Colloids Surf. Physicochem. Eng. Asp. 2021, 622, 126624 10.1016/j.colsurfa.2021.126624.

[ref185] Mastrobattista E.; Crommelin D. J.; Wilschut J.; Storm G. Targeted Liposomes for Delivery of Protein-Based Drugs into the Cytoplasm of Tumor Cells. J. Liposome Res. 2002, 12 (1–2), 57–65. 10.1081/LPR-120004777. 12604039

[ref186] Fujita D.; Suzuki K.; Sato S.; Yagi-Utsumi M.; Yamaguchi Y.; Mizuno N.; Kumasaka T.; Takata M.; Noda M.; Uchiyama S.; Kato K.; Fujita M. Protein Encapsulation within Synthetic Molecular Hosts. Nat. Commun. 2012, 3 (1), 1093 10.1038/ncomms2093. 23033069

[ref187] McConnell S. A.; Cannon K. A.; Morgan C.; McAllister R.; Amer B. R.; Clubb R. T.; Yeates T. O. Designed Protein Cages as Scaffolds for Building Multienzyme Materials. ACS Synth. Biol. 2020, 9 (2), 381–391. 10.1021/acssynbio.9b00407. 31922719

[ref188] Hofweber M.; Dormann D. Friend or Foe—Post-Translational Modifications as Regulators of Phase Separation and RNP Granule Dynamics. J. Biol. Chem. 2019, 294 (18), 7137–7150. 10.1074/jbc.TM118.001189. 30587571 PMC6509508

[ref189] Dignon G. L.; Zheng W.; Kim Y. C.; Mittal J. Temperature-Controlled Liquid–Liquid Phase Separation of Disordered Proteins. ACS Cent. Sci. 2019, 5 (5), 821–830. 10.1021/acscentsci.9b00102. 31139718 PMC6535772

[ref190] Franzmann T. M.; Alberti S. Prion-like Low-Complexity Sequences: Key Regulators of Protein Solubility and Phase Behavior. J. Biol. Chem. 2019, 294 (18), 7128–7136. 10.1074/jbc.TM118.001190. 29921587 PMC6509491

[ref191] Mao A. H.; Crick S. L.; Vitalis A.; Chicoine C. L.; Pappu R. V. Net Charge per Residue Modulates Conformational Ensembles of Intrinsically Disordered Proteins. Proc. Natl. Acad. Sci. U. S. A. 2010, 107 (18), 8183–8188. 10.1073/pnas.0911107107. 20404210 PMC2889596

[ref192] Kapelner R. A.; Obermeyer A. C. Ionic Polypeptide Tags for Protein Phase Separation. Chem. Sci. 2019, 10 (9), 2700–2707. 10.1039/C8SC04253E. 30996987 PMC6419950

[ref193] Dzuricky M.; Rogers B. A.; Shahid A.; Cremer P. S.; Chilkoti A. De Novo Engineering of Intracellular Condensates Using Artificial Disordered Proteins. Nat. Chem. 2020, 12 (9), 814–825. 10.1038/s41557-020-0511-7. 32747754 PMC8281385

[ref194] van Mierlo G.; Jansen J. R. G.; Wang J.; Poser I.; van Heeringen S. J.; Vermeulen M. Predicting Protein Condensate Formation Using Machine Learning. Cell Rep 2021, 34 (5), 108705 10.1016/j.celrep.2021.108705. 33535034

[ref195] Raimondi D.; Orlando G.; Michiels E.; Pakravan D.; Bratek-Skicki A.; Van Den Bosch L.; Moreau Y.; Rousseau F.; Schymkowitz J. *In Silico* Prediction of *in Vitro* Protein Liquid–Liquid Phase Separation Experiments Outcomes with Multi-Head Neural Attention. Bioinformatics 2021, 37 (20), 3473–3479. 10.1093/bioinformatics/btab350. 33983381

[ref196] Pancsa R.; Vranken W.; Mészáros B. Computational Resources for Identifying and Describing Proteins Driving Liquid–Liquid Phase Separation. Brief. Bioinform. 2021, 22 (5), bbaa408 10.1093/bib/bbaa408. 33517364 PMC8425267

[ref197] Kamagata K.; Ariefai M.; Takahashi H.; Hando A.; Subekti D. R. G.; Ikeda K.; Hirano A.; Kameda T. Rational Peptide Design for Regulating Liquid–Liquid Phase Separation on the Basis of Residue–Residue Contact Energy. Sci. Rep. 2022, 12 (1), 13718 10.1038/s41598-022-17829-1. 35962177 PMC9374670

[ref198] Yoshikawa M.; Yoshii T.; Ikuta M.; Tsukiji S. Synthetic Protein Condensates That Inducibly Recruit and Release Protein Activity in Living Cells. J. Am. Chem. Soc. 2021, 143 (17), 6434–6446. 10.1021/jacs.0c12375. 33890764

[ref199] Huber M. C.; Schreiber A.; von Olshausen P.; Varga B. R.; Kretz O.; Joch B.; Barnert S.; Schubert R.; Eimer S.; Kele P.; Schiller S. M. Designer Amphiphilic Proteins as Building Blocks for the Intracellular Formation of Organelle-like Compartments. Nat. Mater. 2015, 14 (1), 125–132. 10.1038/nmat4118. 25362355

[ref200] Shin Y.; Berry J.; Pannucci N.; Haataja M. P.; Toettcher J. E.; Brangwynne C. P. Spatiotemporal Control of Intracellular Phase Transitions Using Light-Activated OptoDroplets. Cell 2017, 168 (1–2), 159–171.e14. 10.1016/j.cell.2016.11.054. 28041848 PMC5562165

[ref201] Garabedian M. V.; Su Z.; Dabdoub J.; Tong M.; Deiters A.; Hammer D. A.; Good M. C. Protein Condensate Formation via Controlled Multimerization of Intrinsically Disordered Sequences. Biochemistry 2022, 61, 2470 10.1021/acs.biochem.2c00250. 35918061 PMC9669173

[ref202] Wensien M.; von Pappenheim F. R.; Funk L.-M.; Kloskowski P.; Curth U.; Diederichsen U.; Uranga J.; Ye J.; Fang P.; Pan K.-T.; Urlaub H.; Mata R. A.; Sautner V.; Tittmann K. A Lysine–Cysteine Redox Switch with an NOS Bridge Regulates Enzyme Function. Nature 2021, 593 (7859), 460–464. 10.1038/s41586-021-03513-3. 33953398

[ref203] Rabe von Pappenheim F.; Wensien M.; Ye J.; Uranga J.; Irisarri I.; de Vries J.; Funk L.-M.; Mata R. A.; Tittmann K. Widespread Occurrence of Covalent Lysine–Cysteine Redox Switches in Proteins. Nat. Chem. Biol. 2022, 18 (4), 368–375. 10.1038/s41589-021-00966-5. 35165445 PMC8964421

